# Succinate-induced macrophage polarization and RBP4 secretion promote vascular sprouting in ocular neovascularization

**DOI:** 10.1186/s12974-023-02998-1

**Published:** 2023-12-21

**Authors:** Tianyi Shen, Ruoyi Lin, Chengyu Hu, Donghui Yu, Chengda Ren, Tingting Li, Meijiang Zhu, Zhongqi Wan, Tu Su, Yan Wu, Wenting Cai, Jing Yu

**Affiliations:** 1 Department of Ophthalmology, Shanghai Tenth People’s Hospital, School of Medicine, Tongji University, Shanghai, 200072 China; 2 Department of Ophthalmology, The Third People’s Hospital of Bengbu, Bengbu, China

**Keywords:** Choroidal neovascularization, Macrophage, Oxygen-induced retinopathy, Succinate, Tip cells

## Abstract

Pathological neovascularization is a pivotal biological process in wet age-related macular degeneration (AMD), retinopathy of prematurity (ROP) and proliferative diabetic retinopathy (PDR), in which macrophages (Mφs) play a key role. Tip cell specialization is critical in angiogenesis; however, its interconnection with the surrounding immune environment remains unclear. Succinate is an intermediate in the tricarboxylic acid (TCA) cycle and was significantly elevated in patients with wet AMD by metabolomics. Advanced experiments revealed that SUCNR1 expression in Mφ and M2 polarization was detected in abnormal vessels of choroidal neovascularization (CNV) and oxygen-induced retinopathy (OIR) models. Succinate-induced M2 polarization via SUCNR1, which facilitated vascular endothelial cell (EC) migration, invasion, and tubulation, thus promoting angiogenesis in pathological neovascularization. Furthermore, evidence indicated that succinate triggered the release of RBP4 from Mφs into the surroundings to regulate endothelial sprouting and pathological angiogenesis via VEGFR2, a marker of tip cell formation. In conclusion, our results suggest that succinate represents a novel class of vasculature-inducing factors that modulate Mφ polarization and the RBP4/VEGFR2 pathway to induce pathological angiogenic signaling through tip cell specialization.

**Graphical Abstract:**

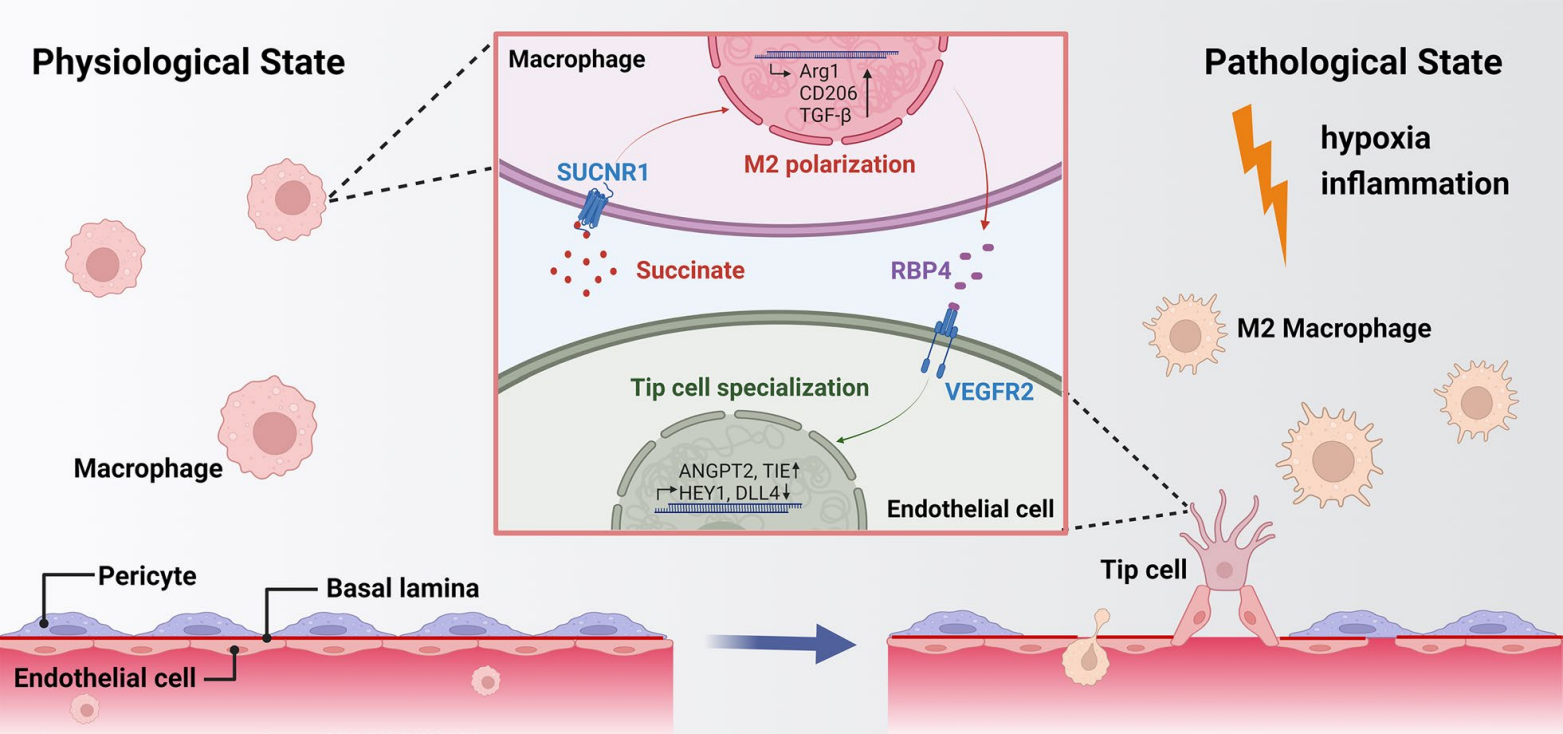

**Supplementary Information:**

The online version contains supplementary material available at 10.1186/s12974-023-02998-1.

## Introduction

Pathological neovascularization is the leading cause of visual impairment in several retinal disorders, including proliferative diabetic retinopathy (PDR), wet age-related macular degeneration (wAMD) and retinopathy of prematurity (ROP). Neovascularization, or angiogenesis, which is defined as new blood vessel formation from existing capillaries [1], appears to be a complicated process involving multiple types of cells. Macrophages (Mφs) are an important cell population in the vascular microenvironment and exert both pro- and anti-angiogenetic effects. Resident Mφs are primarily distributed in tissues or organs and do not migrate in large numbers to specific lesion sites [2]. In the microenvironment of AMD, Mφs from peripheral circulating sources that do not enter the intraocular space in the healthy state are recruited to the CNV region [3]. Mφs can be polarized into classically activated (M1) and alternatively activated (M2) phenotypes, thus influencing immune homeostasis. The high heterogeneity of Mφs is a consequence of intracellular plasticity and the response to different microenvironmental stimuli. M2 polarization has been regarded as a key promoter in various angiogenetic disorders, including choroidal neovascularization (CNV) and retinal neovascularization (RNV) [4, 5]. Increased PGE2 in wAMD participates in M2 polarization and IL-10 production via the EP1R/ PKC signaling pathway, which additionally promotes the proliferation and migration of human choroidal microvascular endothelial cells (HCECs) in vitro [6]. Furthermore, inhibiting the RhoA/ROCK signaling pathway facilitated Mφs to transform from the M2 to the M1 phenotype, reducing vascular leakage and abnormal proliferation in CNV [7]. As a result, in the complex and dynamic intraocular environment, the contribution of the immune system, particularly Mφ recruitment and functional realization, to ocular neovascularization cannot be overlooked.

Cellular crosstalk is based on both direct intercellular contact and indirect communication. Intercellular signaling molecules include cytokines, hormones, neurotransmitters, bioactive molecules, extracellular matrix and cellular metabolites [8]. Cellular energy metabolism underlies most cellular functions, and mounting evidence indicates that endogenous metabolites contribute significantly to both physiological and pathological conditions, including immune homeostasis, tumor development, and neovascularization [9]. In the ocular environment, glucose undergoes metabolism in the retina through the tricarboxylic acid (TCA) cycle and oxidative phosphorylation (OXPHOS), as well as aerobic glycolysis to produce glycerol. After being transported from the choroid to the photoreceptors, glucose converts lactate by optic cells to pyruvate. In response to stimuli such as hypoxic inflammation, this delicate equilibrium is perturbed, resulting in a dysregulated local immune microenvironment [10]. The most well-identified metabolite is lactic acid, which is regarded as a key promoter in cancer immunotherapy, neural excitation, inflammation and angiogenesis [11, 12] and has become a star point because of the identification of lactylation. Aerobic glycolysis leads to high production rates of lactate, which is transported from the subretinal space to the choroidal circulation via the RPE [10]. Song et al. found that the accumulation of lactic acid in the CNV region could affect THP-1 macrophage metabolism and promote angiogenesis in endothelial cells (ECs) [13]. Consequently, it is critical to investigate the levels and potential roles of metabolites in the abnormal neovascular microenvironment, particularly in Mφs function.

Tip cell specialization marks a critical step in angiogenesis and guides other ECs to form vascular networks through chemical signals and cell‒cell interactions. When proangiogenic signals (e.g., the Notch pathway) are triggered, a portion of ECs switch to a tip cell phenotype, extending long filopodia to sense surroundings and mediate the elongation of the vessels, followed by the proliferation of trailing stalk cells to stretch the vascular branches [14]. As tip cells demonstrate high polarity, prominent cellular extensions and the ability to secrete proteins, they can sense and respond to signals from the surrounding environment, including growth factors, extracellular matrix components, and signals from neighboring cells. Yao et al. found that circMET enhanced the interaction between IGF2BP2 and NRARP/ESM1 in oxygen-induced retinopathy (OIR), CNV and proliferative PDR by inhibiting endothelial tip cell specialization [15]. Another study demonstrated that Fibrillin-1 could alter vascular endothelial growth factor (VEGF)/Notch and Smad signaling, thus influencing tip cell identity in retinal vessels [16].

Mφs may regulate angiogenesis by modulating the biological activities of ECs through both direct and indirect regulatory effects. In the immune microenvironment of blood vessels, Spp1 + Mφs are identified in high concentrations in laser-induced CNV eyes and express pro-angiogenic transcriptomes via a variety of pathways, including EC sprouting [17]. In terms of molecular mechanisms, infiltrating monocytes colocalizing with Wnt5a, angiopoietin 1 and Notch-1 were discovered in the vicinity of sprouting spots, which may influence the function of VE-cadherin and interfere with the orderly growth of the vasculature [18]. Although the importance of tip cells is widely accepted, specific markers of endothelial tip cells are lacking. Previous experiments reported that VEGFR2, Kcne3, DLL4 and IGF1R could act as the markers of tip cell specification [19–21] and thus promote neovascularization. Compared with the understanding of ocular angiogenesis, the effects of tip cell specification remain to be elucidated. Accordingly, detecting the role of Mφ in tip cell formation would help in the knowledge of ocular angiogenetic disorders.

Therefore, in the present study, we focused on circulating substances in patients with pathological neovascular disorders by metabolomics. In advanced experiments, we also looked for the influence of a specific metabolite, succinate, on the local immune environment, thus promoting retinal/choroidal vascular outgrowth. As the tip cell is a key but poorly understood point in ocular neovascularization, the role of the secretory factor from Mφ in tip cell specification was analyzed. This study will explain the mechanisms of ocular neovascularization in a multifaceted and joint manner in terms of metabolism, immunity and angiogenesis.

## Materials and methods

### Participant recruitment and aqueous humor (AH) collection

Both wAMD and cataract (CAT) participants were recruited in this research. All AH samples were collected by the same surgeon at the Shanghai Tenth People's Hospital in this single-center study. Patients with cataracts were not accompanied by other ocular diseases. The inclusion criteria of wAMD patients satisfied the diagnosis of clinical classification of AMD [22], categorized as progressive AMD with the presence of CNV on optical coherence tomography (OCT), and no prior intraocular drug injection history. In addition, patients with active ocular inflammation, other retinal diseases, or severe systemic diseases were excluded. Before intravitreal injection or intraocular lens (IOL) implantation, AH samples were acquired in sterile Eppendorf tubes and immediately transferred to − 80 °C for subsequent analysis. Informed consent was obtained from all the participants.

### AH samples preparation and metabolomic analysis

Samples were thawed at room temperature, followed by the addition of isotopic internal standards, and centrifugation was performed for 10 min (4 °C, 12,000 r) to obtain 150 μL of the supernatant for two-part derivatization. After the oximation reaction, *N*,*O*-bis(trimethylsilyl)trifluoroacetamide (BSTFA) and *n*-hexane were added and reacted at 70 °C for 60 min. Samples placed at room temperature were subjected to gas chromatography‒mass spectrometry (GC–MS/MS-Trace 1310/TSQ 9000; Thermo, USA) for detection. TraceFinder 4.1 General Quan software was used to automatically identify and integrate each ion fragment, sequentially calculating the absolute metabolite content based on the peak area and standard curve. Further enrichment and pathway analysis of differentially expressed biomarkers was conducted using MetaboAnalyst 5.0 (http://www.metaboanalyst.ca/) to identify dysregulated pathways in wAMD under Kyoto Encyclopedia of Genes and Genomes (KEGG) database [23].

### Animals

C57BL/6J mice were purchased from Sino-British SIPPR/BK Lab Animal Ltd. (Shanghai, China). They were maintained on a 12-h light/dark cycle and received standard laboratory chow and water under pathogen-free conditions. Mice were euthanized by cervical dislocation under general anesthesia at different time points according to the experimental requirements.

### Animal models and intervention

The mouse CNV model was constructed under the guidance of LeBlanc et al. [24]. In brief, mice were anesthetized by intraperitoneal injection of 4 mL/kg of 1% pentobarbital sodium and topical anesthesia with 0.5% proparacaine, followed by 1 µL of Matrigel (Corning, NY, USA) diluted by phosphate-buffered saline (PBS) (Matrigel:PBS = 1:4) injected using a 34-gauge needle in the temporal side of the subretinal region to induce the formation of CNV. For the group that required succinate added, 1.25 mM succinate (Sigma-Aldrich, USA) was dissolved in PBS in advance. Then, Matrigel and this solution were subsequently mixed at a ratio of 1:4 to produce a mixture with a final concentration of 1 mM succinate. After that, 1 µL of the mixture was injected subretinally for CNV modeling and succinate intervention.

The modeling method of OIR was adapted from Xu et al. [25]. Neonatal C57BL/6J mice and their mothers were exposed to 75% oxygen for 5 consecutive days at P7 and returned to normoxia at P12. Retinal samples were obtained and analyzed at P17.

LV-sh-SUCNR1 virus or negative lentiviral vector (Shanghai Integrated Biotech Solutions Co., Ltd) was administered in the in vivo models. In the CNV model, intravitreal injection of 1 µL lentivirus (10^9^ TU/mL) was given in conjunction with Matrigel induction. In OIR mice, after induction of anesthesia with 4% isoflurane and local anesthesia with 0.5% promethazine, intravitreal injection of 1 µL lentivirus, with or without succinate (1 mM), was performed on P14. Specifically, lentivirus was diluted with PBS to a titer of 10^9^ TU/mL or with succinate to a mixed solution with a final succinate concentration of 1 mM and a lentivirus titer of 10^9^ TU/mL.

### Observation and quantification of neovascularization in vivo

Fluorescein sodium was injected intraperitoneally into anesthetized mice on day 7 after Matrigel induction, and FA images were acquired by confocal scanning laser ophthalmoscopy (Spectralis, Heidelberg Engineering Inc., Heidelberg, Germany) after pupil dilatation by 0.5% Alcaine to observe the extent of CNV leakage [26]. Since ECs of abnormal neovascularization lack mature pericytes for coverage, they have higher permeability than normal vessels, resulting in significant leakage of fluorescein sodium and hyperfluorescence. On day 7 of CNV and P17 of OIR, mice were killed, and eyeballs were fixed in 4% paraformaldehyde for 40 min. Subsequently, intact choroidal or retinal tissues were isolated under the microscope. After permeabilization and blockage, the vessels were labeled overnight with Alexa Fluor 594-conjugated isolectin B4 (IB4, #I21413, Thermo Fisher, USA). After rinsing with PBS, the tissue was spread on a slide and photographed with a fluorescence microscope (Leica Microsystems, Wetzlar, Germany) to capture the vascular morphology. The abnormal vascular areas of CNV and OIR were quantified using ImageJ software. After homogenizing the image scale, the boundaries of the CNVs were delineated using the free selection tool and displayed in square micrometers (μm^2^) [27, 28]. Delineation of neovascular and avascular areas in OIR mice were referred to previous studies [29, 30].

### Electroretinograms (ERGs)

ERG examination was performed on day 7 after Matrigel injection in CNV models and OIR mice using an AVES-2000 electrophysiological apparatus (Kanghuaruiming S&T, Chongqing, China). Referring to the previous method [31, 32], after dark adaptation for 12 h, reference electrode, ground electrode, and golden-ring electrode were attached to the posterior neck, tail, and cornea of the anesthetized mice, respectively. Single flash stimuli (6.325e−2 cd*s/m^2^) were delivered via corneal electrodes, and the monitored potential changes reflected retinal function. The amplitudes of the a- and b-waves were recorded to analyze reactions to brief flashes.

### Histopathological examination

Eyes from each group were fixed in 4% paraformaldehyde, embedded, and sectioned at a thickness of 4 μm parallel to the ocular axis. Sagittal sections were made through the temporal Matrigel-induced position and the center of the ocular axis of the eyes in CNV mice. The eyeballs were stained with hematoxylin–eosin (H&E) to observe histopathological morphology. All photographs were captured under an optical microscope.

### Cell culture and treatment

For Mφ collection, C57BL/6J mice received an intraperitoneal injection of 2 mL of 4% thioglycolate. Forty-eight hours after injection, the peritoneal cavity was flushed with prechilled RPMI 1640 culture medium, and the precipitate was collected by centrifugation and inoculated into culture plates. The medium was changed 2 h later to obtain adherent cells. Human umbilical vein endothelial cells (HUVECs) and endothelial cell growth medium-2 (ECM-2) were provided by Zhongqiao Xinzhou Biotechnology Co., Ltd. (Shanghai, China). All cells were incubated at 37 °C with 5% CO_2_.

According to the experimental protocol, Mφs were incubated with different concentrations of succinate (0.5 mM, 1 mM and 2 mM) for 48 h. LipoRNAiMax (Thermo Fisher, USA) was used to transfect Mφs with negative control siRNA and siRNA oligonucleotides against SUCNR1 (RIBOBIO, Guangzhou, China). Opti-MEM reduced-Serum Medium (Thermo Fisher) was used to dilute LipoRNAiMax and siRNA. The mixed transfection complexes were added uniformly to the Mφs and incubated for 48 h [33]. HUVECs were cultured with treated Mφs in 0.4-μm pore size co-culture plates (Corning, NY, USA) to observe angiogenic function with reference to the previous method [34]. In brief, intervened Mφs were seeded in the upper chamber and co-cultured for 48 h with HUVECs in the lower chamber before performing functional studies on HUVECs.

### Mφ identification and flow cytometry analysis

After the intervention, Mφs were collected and incubated with anti-F4/80-APC/Cy7 (BioLegend), anti-CD11b-FITC (BioLegend), anti-CD86-PE (BioLegend), and anti-CD206-APC (BioLegend) antibodies. Flow cytometry was performed on an FACSCalibur (BD Biosciences) and analyzed using FlowJo software. The gating strategy for M2 polarization was as follows: the CD11b^+^- and F4/80^+^-populations were gated first, and then the CD11b^+^ F4/80^+^ population was further gated based on CD206 intensity.

### Examination of HUVEC function

Cell viability assays were performed based on the instructions of the CCK-8 kit (Yeasen, Shanghai, China). Briefly, 10 μL/well of CCK-8 reagent was incubated for 2 h, and the optical density (OD) at 450 nm was measured by a spectrophotometer. Cell nuclei were labeled with an EdU staining kit (Yeasen, Shanghai, China). Photographs were taken with a fluorescence microscope, and the percentage of EdU-positive cells was calculated by ImageJ.

Cell migration ability was detected using wound healing and Transwell assays. Wounds were applied on proliferating cells using a 200-μL pipette tip. At 0, 6, 12 and 24 h after scratch formation, images were taken at the same location to calculate the cell migration rate. Transwell experiments were performed on Transwell plates (8 μm, Corning, USA). HUVECs were placed in the upper chamber (serum-free medium). After 20 h, cells that migrated to the lower chamber (complete medium) were stained using crystalline violet. After splitting the stacks of cells, particles were counted in three random areas for enumeration under a light microscope using ImageJ software following the guidance of Pijuan et al. [35].

Co-cultured HUVECs were analyzed for cellular tube-forming ability. Matrigel was incubated on 96-well plates at 37 °C for half an hour, followed by inoculation of cells, and capillary-like structures were recorded after 6 h. We calculated the number of junctions and branches, as well as the total tube length and increase of tube formation in the different groups. Increase of tube formation (%) was calculated as follow: total tube length treatment /total tube length control × 100% [36, 37]. Three replicates of each group were used to calculate the mean number of tubules formed.

### RNA sequencing and bioinformatics analysis

Total RNA was extracted from normal and pretreated (1 mM succinate for 48 h) mouse peritoneal Mφs using TRIzol reagent. RNA libraries were sequenced on the Illumina NovaSeqTM 6000 platform from OE Biotech, Inc. Each sample gene count was normalized using DESeq2 software [38] to screen for genes with a fold-change greater than 2. Subsequently, the screened differentially expressed genes were analyzed for GO and KEGG pathways.

### Co-immunoprecipitation assays to identify extracellular VEGFR2-RBP4 interactions

To perform co-immunoprecipitation (Co-IP), exogenous human RBP4 protein was applied to HUVECs. According to an acknowledged Co-IP protocol [39], an RBP4 antibody against RBP4 was coupled to Sepharose beads through protein A/G (BioTNT, L-1008 A). Ultimately, the complexes containing RBP4 and binding proteins were immunoprecipitated with RBP4 antibody-coupled beads by centrifugation followed by western blotting for visualization (using VEGFR2 (Proteintech, 26415-1-AP)).

### Choroidal sprouting assay

After euthanasia, the eyeballs of 4-week-old C57BL/6J mice were removed and transferred to prechilled 1640 medium containing 10% FBS. The choroidal explants containing the RPE/choroid/sclera complex in the peripheral region were separated and cut into 1 × 1 mm^2^ pieces. The explants were immediately embedded in Matrigel in 24-well plates containing 500 μL of 1640 medium. Photographs were taken under a microscope on day 4, and the germination area was measured using ImageJ software, based on a previous method [40]. The threshold function was used to set the vascular sprout boundary, and then the choroidal tissue region was removed to calculate the sprouting area.

### RNA extraction and qPCR

TRIzol reagent (Thermo Fisher Scientific) was used to extract total RNA, and cDNA was synthesized using a HiScript III cDNA synthesis kit (Vazyme, China). QPCR was conducted by ChamQ Universal SYBR qPCR Master Mix (Vazyme, China). The relative abundance of target genes was evaluated by 2^−ΔΔCt^. Primer sequences utilized for qPCR analysis of genes are listed in Additional file 1: Table S1.

### Western blotting

Total proteins from tissues and cells were collected on ice using RIPA lysis buffer, and protein concentrations were quantitated based on the OD value at 562 nm of the BCA standard and target proteins. Equal protein aliquots (30 μg) were loaded on 7.5–15% polyacrylamide gels and transferred to nitrocellulose membranes. After blocking the membrane at room temperature in 5% nonfat milk, proteins were incubated with primary antibodies at 4 °C overnight. Subsequently, the bands were combined with specific secondary antibodies for 1 h, and the protein bands were scanned using an Odyssey system (LI-COR). Antibodies specific for Arg1 (1:2000, #66129-1-Ig), TGF-β (1:1000, #21898-1-AP), TNFα (1:1000, #17590-1-AP), IL6 (1:1000, #66146-1-Ig), VEGF-A (1:1000, #19003-1-AP), CD31 (1:1000, #66065-2-Ig), RBP4 (1:1000, #11774-1-AP), VEGFR2 (1:1000, #26415-1-AP), and β-tubulin (1:2000, #10094-1-AP) were purchased from Proteintech (Rosemont, IL, USA). Antibody specific for MMP2 (1:500, #GB11130-100) was purchased from Servicebio (Wuhan, China). Antibody specific for SUCNR1 (1:1000, #NBP1-00861) was purchased from Novus Biologicals (Littleton, Colorado, USA).

### Immunofluorescence staining

Fixed cells were antigen blocked in 5% BSA and 0.1% Triton X-100. Cells were incubated with primary antibodies (anti-VEGF-A, anti-CD31, anti-Arg1, anti-TGF-β and anti-VEGFR2) overnight and immersed in fluorescent secondary antibodies for 40 min. Antibodies specific for VEGF-A (1:200, #19003-1-AP), CD31 (1:200, #66065-2-Ig), Arg1 (1:200, #16001-1-AP), TGF-β (1:200, #21898-1-AP) and VEGFR2 (1:200, #26415-1-AP) were purchased from Proteintech. Finally, nuclei were stained with DAPI. Confocal results were captured on an LSM 980 Airyscan SR microscope (Carl Zeiss, Micro Imaging GmbH, Jena, Germany).

### Enzyme-linked immunosorbent assay (ELISA)

Cell-conditioned medium (CM) and intracellular proteins were extracted from normal and pretreated Mφs (0.5, 1 and 2 mM succinate for 48 h). For intracellular protein extraction, Mφs were collected and resuspended in PBS at a cell concentration of approximately 10^6^ cells/mL. Subsequently, the cells were sonicated to disrupt cellular structures and then centrifuged at 8000 rpm for 20 min at 4 °C to remove cellular debris. Following the protocol of the instructions, ELISA kits, including RBP4 (#U96-1932E, YOBIBIO), TNFα (#U96-3112E, YOBIBIO), iNOS (#U96-3446E, YOBIBIO) and IL6 (#U96-1511E, YOBIBIO), were employed to detect the protein levels. Eventually, the optical density (OD) of the samples was measured at 450 nm using a microplate reader (Molecular Devices, SpectraMax iD5, California, USA).

### Statistical analysis

All data were statistically analyzed using GraphPad Prism 9. A t test was used for comparisons between two groups, and one-way ANOVA was used for multigroup comparisons. All data are expressed as the mean ± standard deviation (mean ± SD). *P* < 0.05 was considered a statistically significant difference. All experiments were repeated at least three times.

## Results

### Activation of the TCA cycle and increased succinate production detected in AH of wAMD patients

**Fig. 1:**
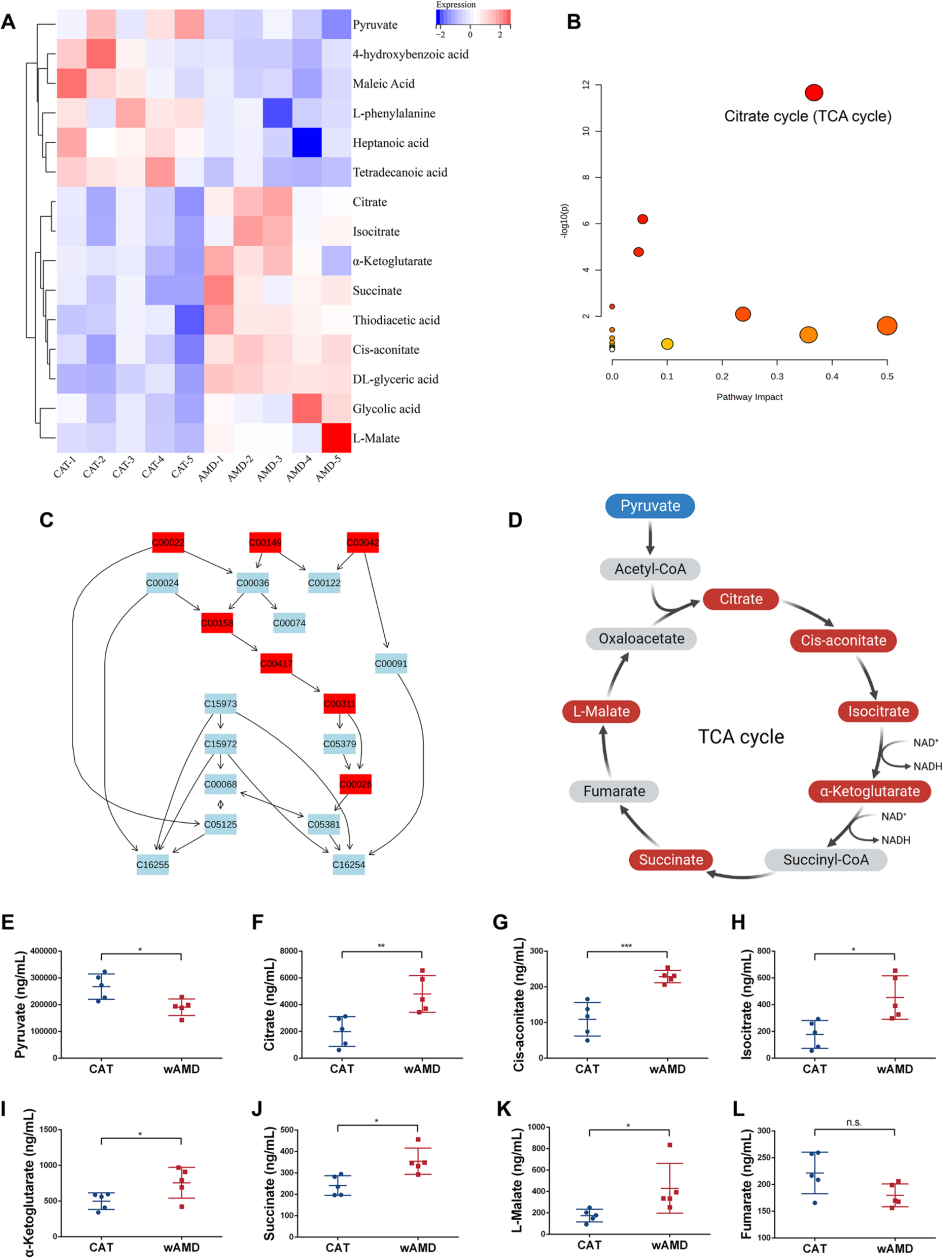
Metabolic analysis between the CAT group and wAMD group. **A** Heatmap analysis between the two groups. **B** Metabonomic pathway analysis. **C** Different metabolites in the TCA cycle according to the KEGG database. **D** Display of meaningful metabolites in the TCA cycle. Red represents elevated expression, and blue represents decreased expression. **E**–**L** Specific concentrations of pyruvate, citrate, *cis*-aconitate, isocitrate, α-ketoglutarate, succinate, l-malate and fumarate (*n* = 5). *P < 0.05, **P < 0.01, ***P < 0.001

Ten participants were recruited in this research, including five patients with wAMD and five patients with CAT as controls. Basic information and ophthalmic examinations of the recruited patients are available in Additional file 1: Fig. S1. The difference in age, sex and intraocular pressure (IOP) between the control group and wAMD patients showed no statistical significance (Additional file 1: Fig. S1A). Fundus photography and macular OCT showed hemorrhagic exudation in the macula with subretinal fluid and CNV morphology in patients with wAMD (Additional file 1: Fig. S1B). A total of 15 metabolites with significant expression changes were detected, of which 9 were upregulated and 6 were downregulated. Heatmap analysis depicted differentially expressed metabolites in the two groups of AH (Fig. 1A). Metabonomic pathway analysis (MetPA) based on the KEGG database detected that the TCA cycle was significantly enriched and contained 7 metabolites (Fig. 1B, C). Figure 1D displays the meaningful metabolites we measured in the wAMD group of the TCA cycle, with blue color denoting a decrease in expression and red signifying an increase. In wAMD patients, pyruvate was significantly decreased, while the levels of citrate, cis-aconitate, isocitrate, α-ketoglutarate, succinate and l-malate were upregulated (Fig. 1E–K). It is possible that the decrease in fumarate could be attributed to downstream depletion of l-malate, although the difference was not significant (Fig. 1L). The evidence indicated that the TCA cycle was abnormally activated and that succinate production was promoted in wAMD patients.

### Enrichment of SUCNR1 and Arg1 in animal models of CNV and OIR

**Fig. 2:**
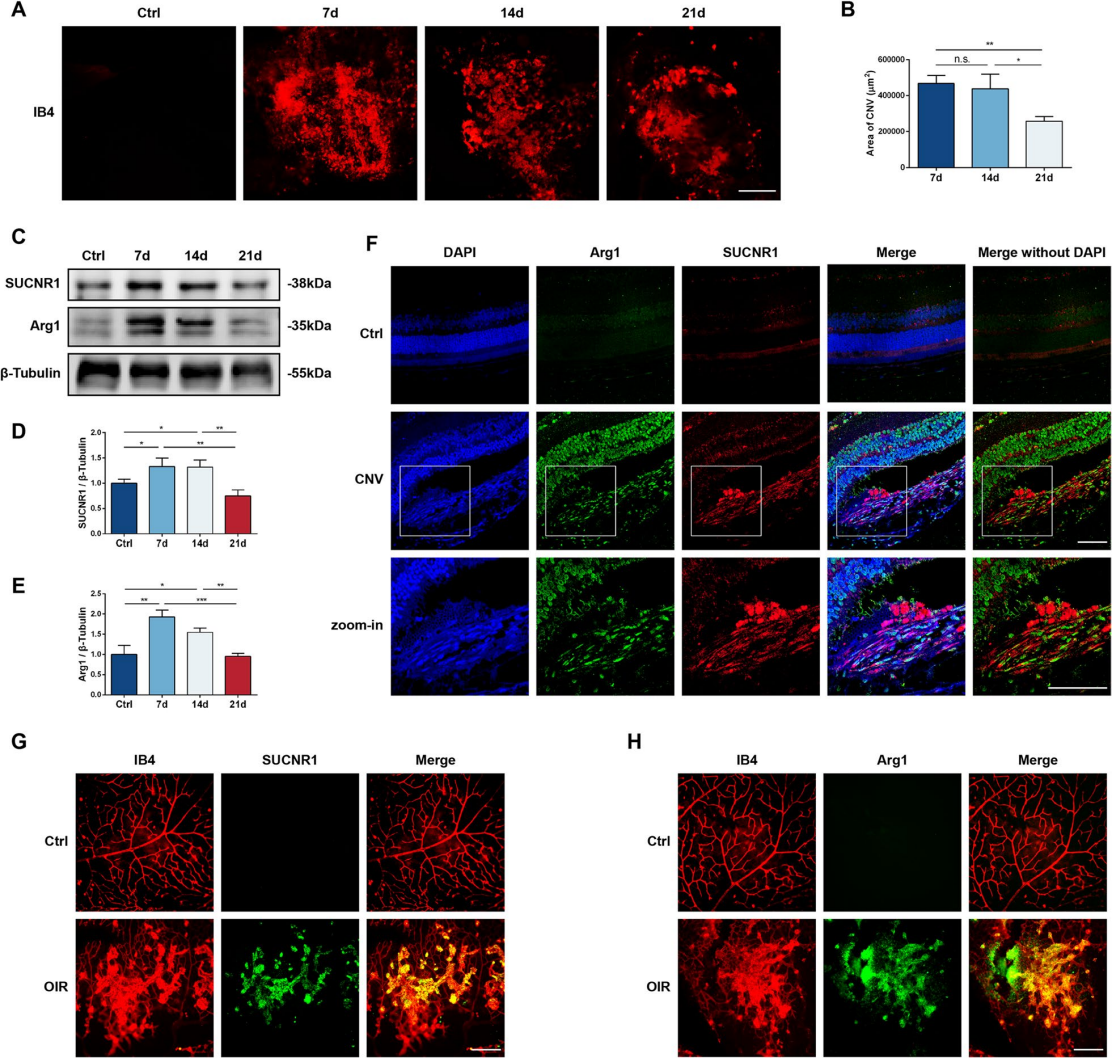
Relative protein expression in animal CNV and OIR models. **A**, **B** Choroidal IB4 staining and areas of CNV in control and on days 3, 7 and 14 after Matrigel induction. Scale bar: 200 μm (*n* = 3). **C**, **E** Relative expression of SUCNR1 and Arg1 in control and CNV animals (*n* = 3). **F** Immunofluorescence staining of DAPI (blue), Arg1 (green) and SUCNR1 (red) in control and CNV eyes. Scale bar: 100 μm. **G**, **H** Immunofluorescence staining of IB4, SUCNR1 and Arg1 in the retinas of control and OIR models. Scale bar: 200 μm. *P < 0.05, **P < 0.01, ***P < 0.001

To further investigate metabolic dysregulation in ocular neovascular diseases, Matrigel-induced mouse CNV models and OIR mice were implemented. FA (Additional file 1: Fig. S2) and choroidal IB4 (Fig. 2A, B) flat mount staining displayed a significant appearance of CNV on day 7 after modeling, accompanied by sodium fluorescein leakage. The CNV area on day 14 remained similar to that on day 7 and decreased from day 21. Interestingly, the expression levels of the succinate receptor SUCNR1 and the M2 marker Arg1 were both positively correlated with CNV size, which was significantly detected on day 7 and remained elevated until day 14 (Fig. 2C–E). Colocalization of SUCNR1 and Arg1 in the CNV region was observed; thus, M2 Mφs could be regarded as a key receptor of succinate (Fig. 2F). In addition, SUCNR1 and Arg1 expression was significantly elevated in the abnormal neovascular lesions in retinal samples from OIR mice at P17 (Fig. 2G, H). Consequently, both chemotaxis of M2 polarization of Mφs and succinate accumulation were implicated in pathological neovascularization.

### Succinate promoted M2 polarization of Mφs in vitro

**Fig. 3:**
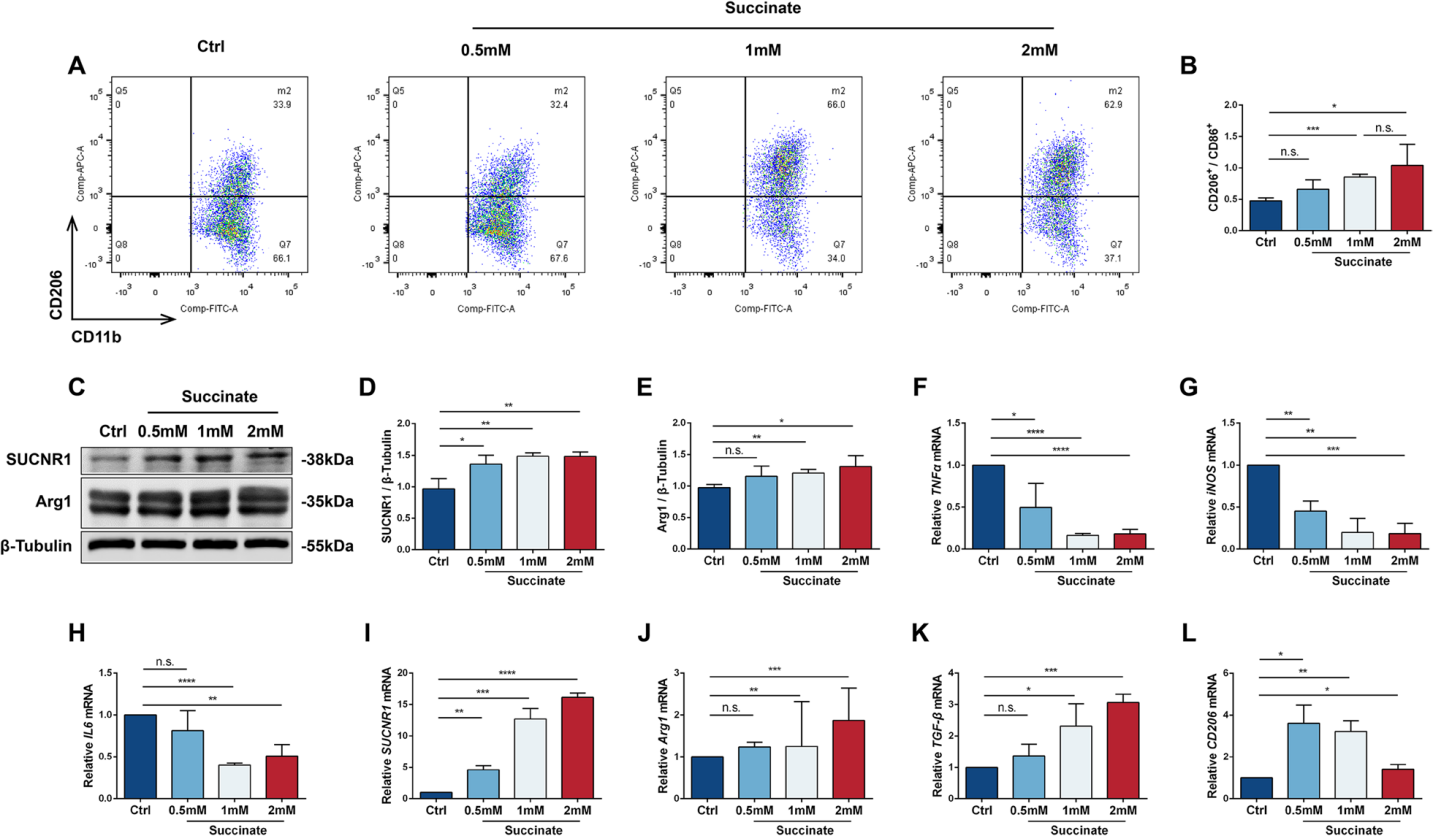
Mφ phenotypes after induction with different concentrations of succinate (0, 0.5 mM, 1 mM and 2 mM for 48 h). **A** Flow cytometry analysis of Mφs after treatment with different concentrations of succinate. **B** The ratio of CD206^+^/CD86^+^ cells in Mφs in the four groups (*n* = 3). **C**–**E** Relative protein levels of SUCNR1 and Arg1 in the four groups (*n* = 3). **F**–**L** Relative RNA expression of TNFα, iNOS, IL6, SUCNR1, Arg1, TGF-β and CD206 (*n* = 3). *P < 0.05, **P < 0.01, ***P < 0.001, ****P < 0.0001

To further verify the effect of succinate treatment on Mφ polarization, advanced analyses based on cultured peritoneal Mφs were conducted. Mφs without succinate induction served as a control group and were compared with Mφs treated with different concentrations of succinate (0.5 mM, 1 mM and 2 mM) for 48 h. The F4/80^+^CD11b^+^-Mφs were gated, and then the proportions of CD206^+^ (M2-type marker) and CD86^+^ (M1-type marker) cells were evaluated. The rates of CD206^+^/CD86^+^ Mφs were significantly increased after succinate intervention, indicating that succinate promoted M2 polarization (Fig. 3A, B). Exogenous succinate dose-dependently promoted the expression of SUCNR1 at both the mRNA (Fig. 3I) and protein levels (Fig. 3C, D) while simultaneously inducing Arg1 production (Fig. 3C, E). In addition, the transcriptional levels of M1-type-related genes (TNFα, iNOS and IL6) (Fig. 3F–H) as well as protein levels (Additional file 1: Fig. S3) diminished after a certain concentration of succinate intervention, accompanied by the upregulation of M2-type-related gene (Arg1, TGF-β and CD206) expression (Fig. 3J–L).

### Succinate mediated M2 polarization via SUCNR1

**Fig. 4:**
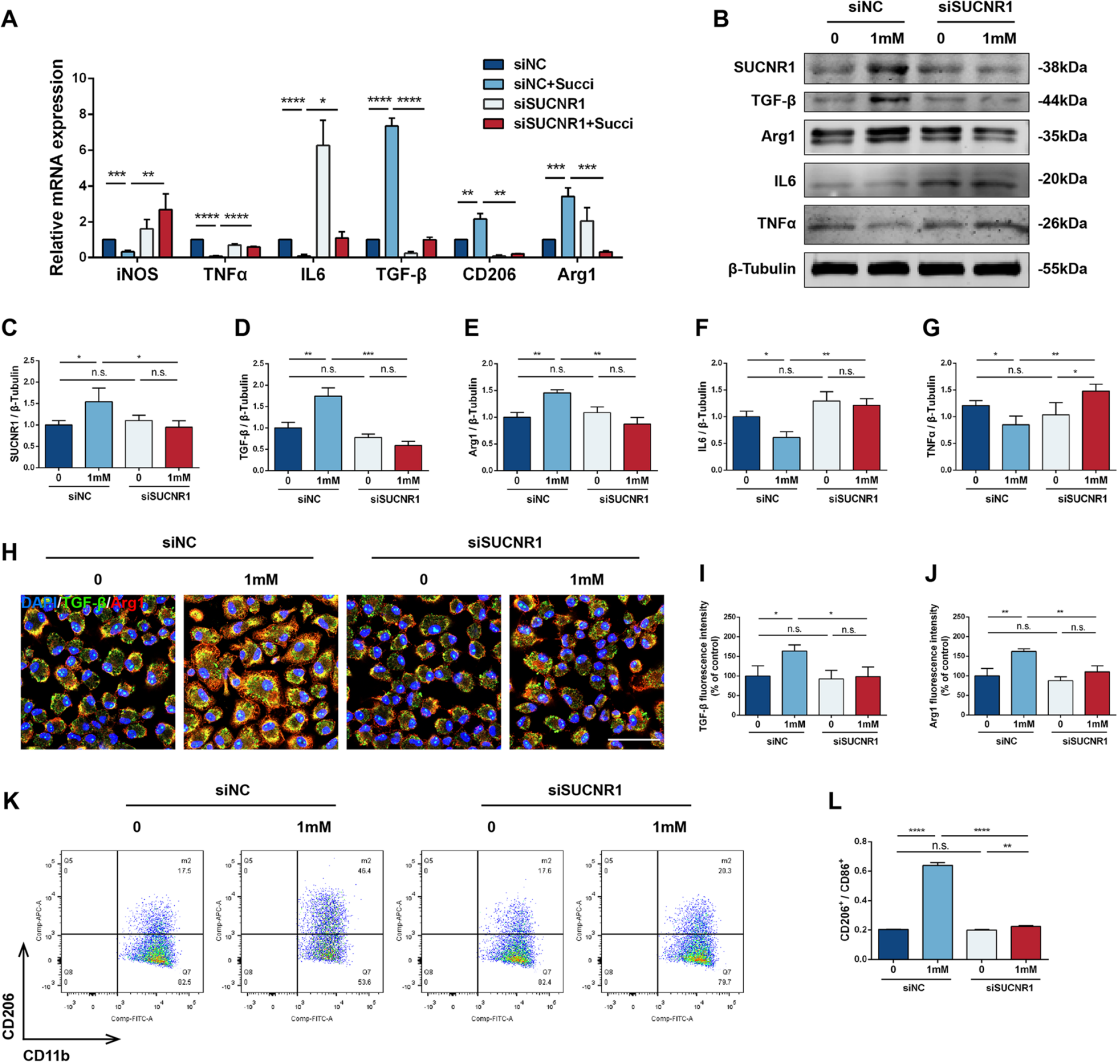
Effect of SUCNR1 inhibition on the Mφ phenotype. Mφs were treated with siRNAs and 1 mM succinate for 48 h and divided into four groups: siNC, siNC + succinate, siSUCNR1 and siSUCNR1 + succinate. **A** Relative RNA expression of iNOS, TNFα, IL6, TGF-β, CD206 and Arg1 in the four groups (*n* = 3). **B**–**G** Relative protein levels of SUCNR1, TGF-β, Arg1, IL6 and TNFα in the four groups (*n* = 3). **H** Immunofluorescence staining of Mφs with DAPI (blue), TGF-β (green) and Arg1 (red). Scale bar: 50 μm. **I**, **J** Relative fluorescence intensity of TGF-β and Arg1 (*n* = 3). **K** Flow cytometry analysis of Mφs in the four groups. **L** The ratio of CD206^+^/CD86^+^ cells in Mφs (*n* = 3). *P < 0.05, **P < 0.01, ***P < 0.001, ****P < 0.0001

To investigate whether succinate promotes Mφ polarization via SUCNR1, an in vitro SUCNR1 knockdown intervention was conducted. Primary peritoneal Mφs were transfected with siNC or siSUCNR1 and cultured with succinate (1 mM, 48 h) as needed. The application of siSUCNR1 inhibited the expression of Arg1, TGF-β and CD206, while facilitating the levels of TNFα, iNOS and IL6 mRNA after exogenous administration of succinate (Fig. 4A). Furthermore, siSUCNR1 decreased the protein expression of SUCNR1, Arg1 and TGF-β after succinate induction and attenuated the inhibitory effects of succinate on TNF-α and IL6 (Fig. 4B–G). Immunofluorescence results showed an enrichment of Arg1 and TGF-β in Mφs after succinate interventions and a morphological transition from spherical to shuttle-shaped cells with increased tentacles, which was inhibited by siSUCNR1 (Fig. 4H–J). Succinate-mediated upregulation of F4/80^+^CD11b^+^CD206^+^ Mφs was blocked by siSUCNR1 treatment (Fig. 4K, L). The above results indicated that succinate could stimulate M2 polarization through SUCNR1.

### Succinate-induced polarized Mφs enhanced HUVEC migration and proliferation

**Fig. 5:**
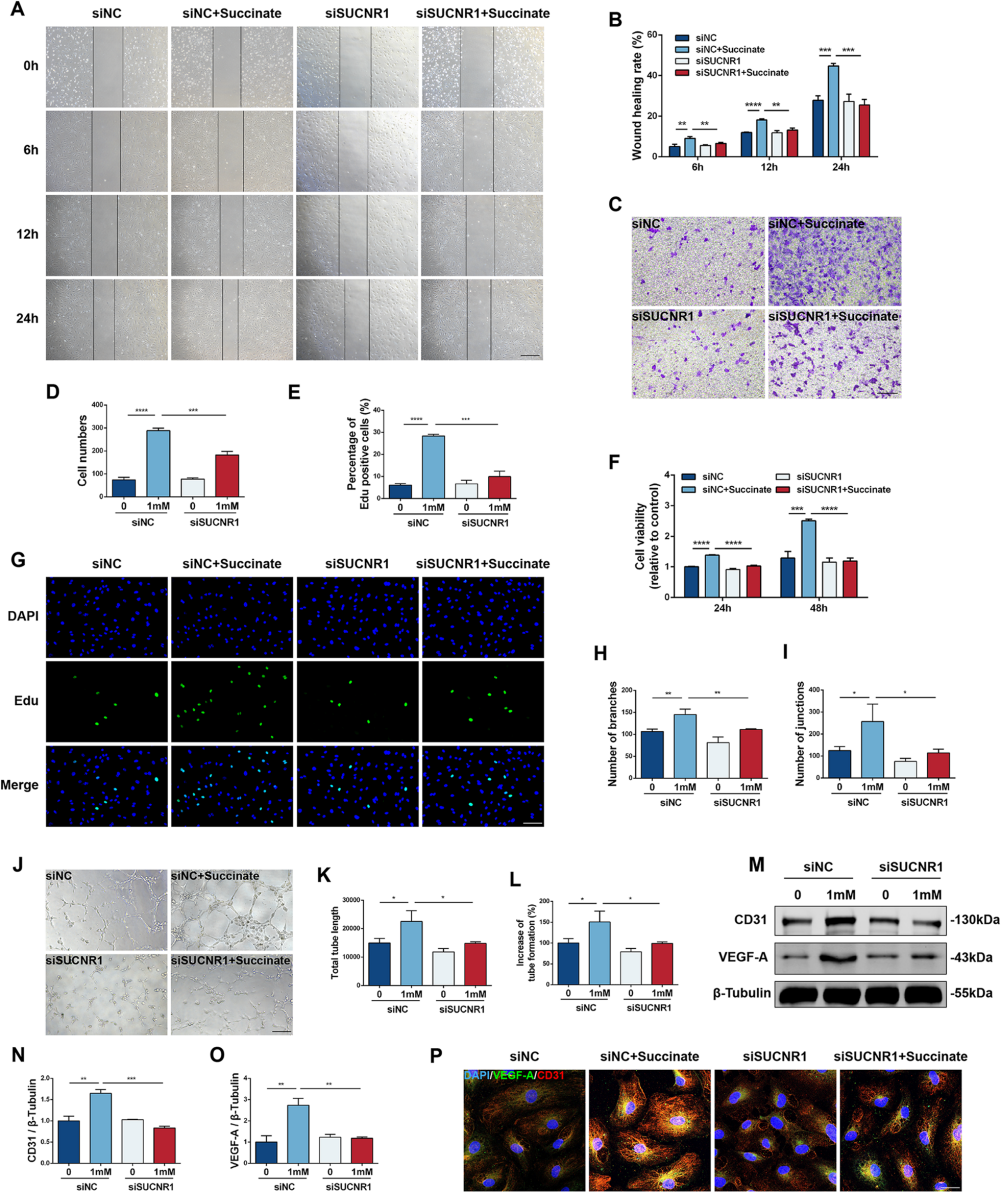
Migration, proliferation and angiogenesis ability of HUVECs co-cultured with four groups of Mφs (siNC, siNC + succinate, siSUCNR1 and siSUCNR1 + succinate). **A**, **B** Images were measured at 0 h, 6 h, 12 h and 24 h in the scratch wound healing test. The cell migration rate was used to indicate migratory ability as described in the article. Scale bar: 200 μm (*n* = 3). **C**, **D** In the Transwell assay, ImageJ software was used to calculate the stained cells in the four groups. Scale bar: 200 μm (*n* = 3). **E**, **G** EdU assays revealed the proliferation of HUVECs, and the percentage of EdU-positive cells in the four groups was calculated. Scale bar: 200 μm (*n* = 3). **F** Cell viability was tested by CCK8 assay, and the OD at 450 nm was recorded to calculate cell viability at 24 h and 48 h (*n* = 3). **J** Tubular formation of four groups was recorded after being cultured on Matrigel for 6 h. Scale bar: 200 μm. The number of branches (**H**), junctions (**I**), total tube length (**K**) and increase of tube formation (%) (**L**) were qualified by ImageJ software (*n* = 3). **M**–**O** Relative protein levels of CD31 and VEGF-A in the four groups (*n* = 3). **P** Immunofluorescence staining for DAPI (blue), VEGF-A (green) and CD31 (red) in HUVECs. Scale bar: 50 μm. *P < 0.05, **P < 0.01, ***P < 0.001, ****P < 0.0001

Since the Mφ phenotype and vascular endothelial cell migration are critically interrelated, it is necessary to evaluate the HUVEC function mediated by succinate-induced Mφs. Mφs, pretreated with siRNA and 1 mM succinate for 48 h, were co-cultured with HUVECs, and the migration capacity of HUVECs was assessed by wound healing and Transwell assays. Results revealed that succinate-induced Mφs enhanced HUVEC scratch repair and migration compared to the control medium, whereas siSUCNR1 impaired Mφ polarization-mediated HUVEC migration (Fig. 5A–D). Furthermore, we performed interventions using hydroxyurea (200 mM, 24 h) and succinate-induced Mφs on HUVECs to exclude the effect of proliferation. The scratch migration rate and transwell capacity in HUVECs, which were co-cultured with succinate-induced Mφs as well as hydroxyurea, were increased compared to those in the control group, indicating that the change in HUVEC function was caused by migration rather than proliferation (Additional file 1: Fig. S4).

In addition, CCK8 and EdU staining were performed to analyze the proliferative capability of HUVECs. Mφs treated with succinate for 48 h were sufficient to increase the viability of HUVECs and to increase the number of cells in the DNA-synthesizing phase. Notably, after transient transfection with siSUCNR1, polarized Mφs significantly suppressed the viability and number of EdU-positive cells in HUVECs (Fig. 5E–G). Overall, succinate-induced Mφs promoted HUVEC proliferation and migration, whereas SUCNR1 knockdown impaired HUVEC function while inhibiting M2 polarization.

### Succinate-induced Mφs promoted tube-forming capacity and expression of pro-angiogenetic genes in HUVECs

Migration of vascular endothelial cells into tubular structures represents an integral process in neovascularization. After succinate induction, Mφs stimulated HUVECs toward a tubular orientation, whereas siSUCNR1 attenuated the number of tubular branches and junctions, total tube length, and the increase of tube formation (%) (Fig. 5H–L). At the molecular level, we examined the protein levels of CD31 and VEGF-A, which are typically recognized as vascular function-associated proteins, in HUVECs. As shown in Fig. 5M–P, stimulation of M2-polarized Mφs was responsible for increased expression of CD31 and VEGF-A, and their fluorescence intensities were improved in HUVECs, which could be reversed by suppression of SUCNR1 in Mφs. Furthermore, VEGFR2 protein expression in HUVECs was promoted by succinate-induced Mφs and decreased after SUCNR1 inhibition (Additional file 1: Fig. S5). However, the protein level of MMP2 was not significantly regulated after succinate intervention, suggesting that Mφs may influence the function of ECs through VEGFR2 rather than the MMP2 pathway (Additional file 1: Fig. S5). In all, succinate-induced M2-polarized Mφs exhibited proangiogenic potential in vitro.

### Succinate mediated M2 polarization and fundus neovascularization in vivo via SUCNR1

**Fig. 6:**
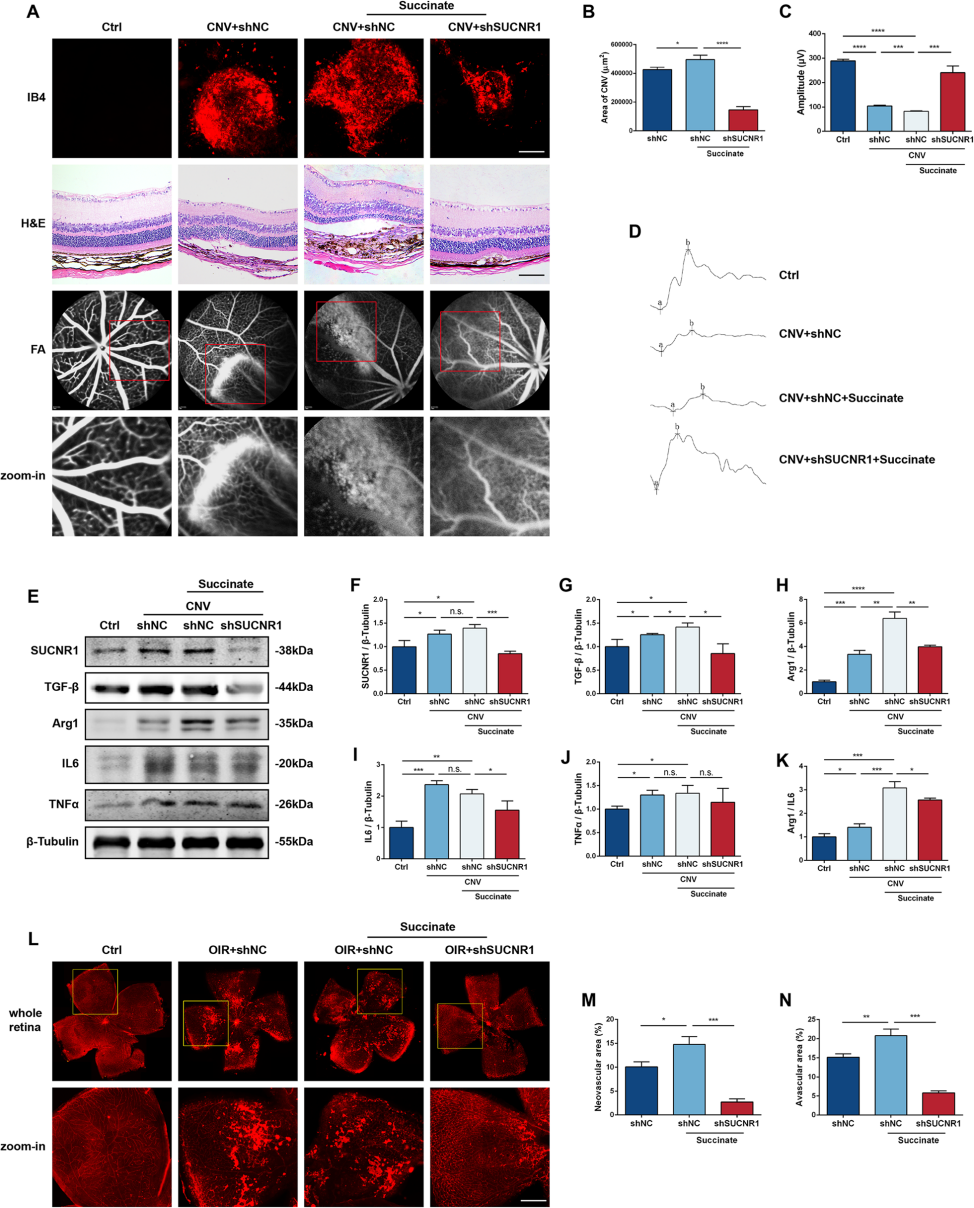
Knockdown of SUCNR1 reduced the severity of succinate-induced CNV and RNV in animals. C57 mice were randomly divided into four groups: control, CNV + shNC, CNV + shNC + succinate, and CNV + shSUCNR1 + succinate. (**A**, top row) **B** Choroidal IB4 staining and areas of CNV in four groups on day 7 after Matrigel induction. Scale bar: 200 μm (*n* = 3). (**A**, second row) H&E-stained sections of eyeballs. Scale bar: 100 μm. (**A**, third and fourth row) FA images of four groups. **C**, **D** Waveform and amplitude of ERGs in each group (*n* = 3). **E**–**J** Protein levels of SUCNR1, TGF-β, Arg1, IL6 and TNFα in animals (*n* = 3). **K** The Arg1/IL6 protein level ratio (*n* = 3). **L**–**N** Retinal IB4 staining and size of neovascular area as well as avascular area in OIR models. Scale bar: 400 μm (*n* = 3). *P < 0.05, **P < 0.01, ***P < 0.001, ****P < 0.0001

To ascertain whether succinate leads to Mφ polarization and neovascularization in vivo, we constructed CNV and OIR mouse models. As shown in Fig. 6A, choroidal flat mount staining on day 7 after Matrigel induction marked a significant IB4-positive area in the CNV models. Moreover, local administration of succinate increased the vascular areal extents, and HE staining reflected an augmentation in the thickness of neovascularization. In contrast, shSUCNR1 injection limited the extent of CNV and attenuated sodium fluorescein leakage under FA (Fig. 6A, B), effectively mitigating the adverse effects of succinate on retinal function (Fig. 6C, D). Compared with that in OIR mice, succinate further expanded the area of abnormal retinal neovascularization and avascular area, while shSUCNR1 improved the physiological structure of retinal vessels (Fig. 6L–N). In addition, the shSUCNR1 + succinate group of OIR mice had higher vascular density than the control group, which may be related to the abnormal vascular pruning of the retina due to OIR modeling [36]. In terms of retinal function, the inhibition of SUCNR1 significantly attenuated further retinal function impairment caused by succinate injection in OIR mice (Additional file 1: Fig. S6).

Compared with the CNV group, exogenous succinate promoted SUCNR1 expression, while shSUCNR1 effectively inhibited SUCNR1 levels compared with the CNV group (Fig. 6E, F). The expression of the M2 polarization-related proteins Arg1 and TGF-β was further elevated after succinate intervention and downregulated after SUCNR1 inhibition (Fig. 6G, H). Interestingly, the levels of inflammatory factors, including IL6 and TNFα, were synchronously increased in the CNV group and maintained or slightly decreased after succinate and shSUCNR1 interventions, which potentially correlated with the increase in the total number of Mφ due to recruitment in CNV mice (Fig. 6I, J). Therefore, the ratio of Arg1/IL6 in different groups was analyzed to provide a reference guide for the possible direction of Mφ polarization. The results demonstrated that the Arg1/IL6 ratio was enhanced in CNV mice compared with normal mice, and the injection of succinate further increased the Arg1/IL6 ratio, which was diminished by shSUCNR1 (Fig. 6K). Collectively, these observations support the notion that succinate influences the M2 polarization of Mφs and thereby promotes aberrant neovascularization in vivo via the SUCNR1 pathway.

### Alterative secretome gene profile of Mφs treated with succinate

**Fig. 7:**
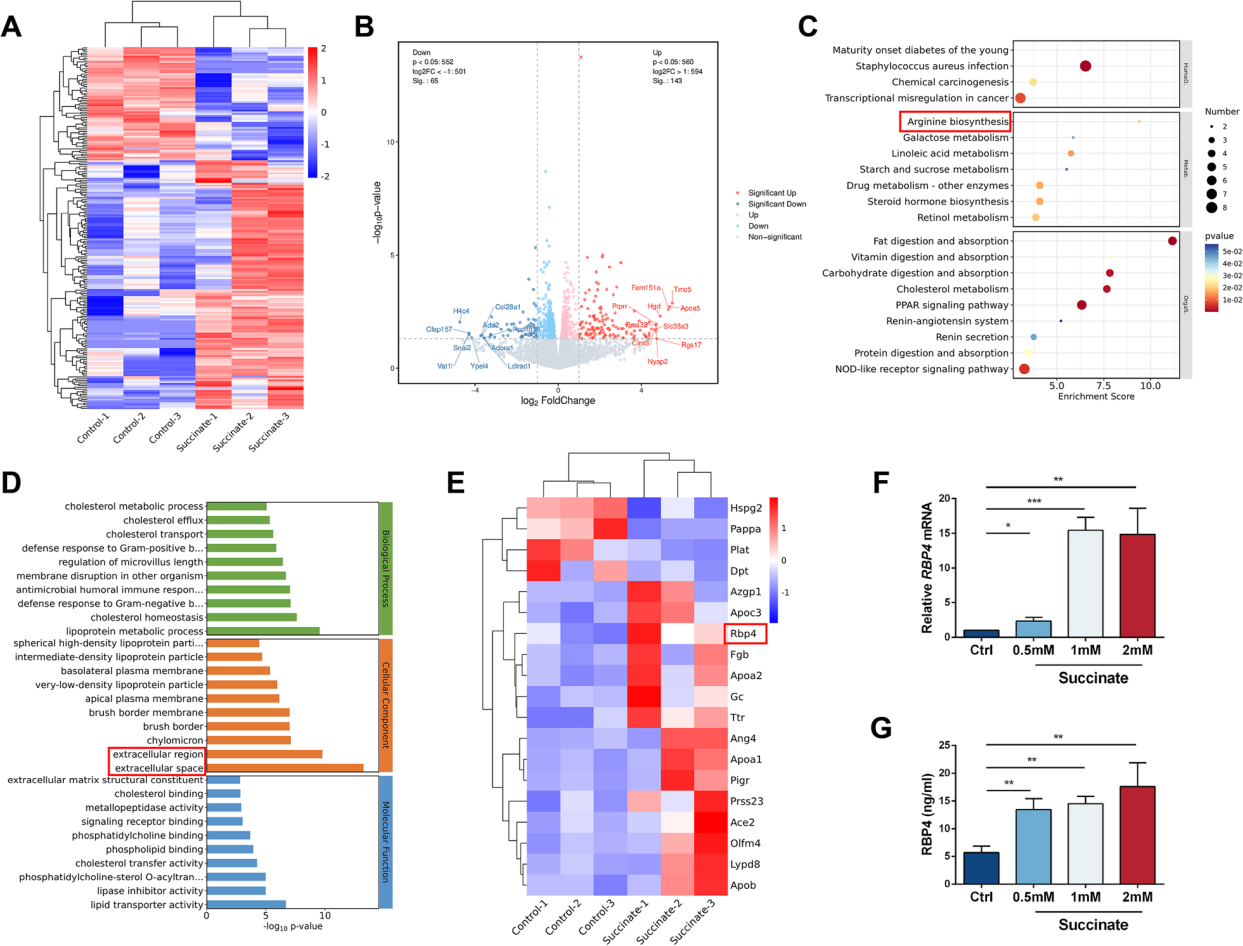
Analysis of gene expression profiles between control and succinate (1 mM for 48 h) groups in Mφs. **A** Heatmap of different genes in the two groups. **B** Log2-fold change for succinate treatment compared to the control. **C** KEGG pathway enrichment analysis of the top 20 differentially expressed pathways. **D** GO analysis of the top 10 differentially expressed pathways in the BP, CC and MF categories. **E** Heatmap of significantly different secreted genes. **F** Relative mRNA expression of RBP4 in succinate-treated Mφs (*n* = 3). **G** RBP4 concentration in Mφ supernatant after succinate intervention (*n* = 3). *P < 0.05, **P < 0.01, ***P < 0.001

To further determine the mechanism of the effect of succinate intervention in Mφs to promote angiogenesis, control and succinate-treated (1 mM, 48 h) Mφs were subjected to RNA sequencing analyses. Compared with the control group, 208 differentially expressed genes were detected in Mφs after succinate intervention, among which 143 genes were upregulated and 65 genes were downregulated (Fig. 7A, B). KEGG pathway enrichment analyses revealed that arginine biosynthesis was activated after succinate intervention, consistent with previous findings of Arg1 enrichment after succinate-induced M2 polarization in Mφs (Fig. 7C). Gene Ontology (GO) analysis was conducted to analyze the enrichment of differential genes at the level of biological process (BP), cellular component (CC), and molecular function (MF) (Fig. 7D). The data revealed the enrichment of differential genes in extracellular regions and extracellular space. Due to the results of in vitro fluorescence staining and co-culture, we hypothesized that it might be possible that genes with secretory roles in Mφs activate EC functions. To further identify key genes secreted by Mφs affecting neovascularization, we compared the gene list with the UniProt database of mice with secretory potential genes (https://www.uniprot.org/) and finally screened 19 differentially expressed secreted genes (15 with increased expression and 4 with decreased expression) (Fig. 7E). Based on fold change and gene expression combined with ELISA and qPCR analysis (Fig. 7F, G), we selected retinol binding protein 4 (RBP4) as a secreted factor by polarized Mφs after succinate intervention that affects vascular endothelial cell function.

### RBP4 induced endothelial tip cell specialization through VEGFR2

**Fig. 8:**
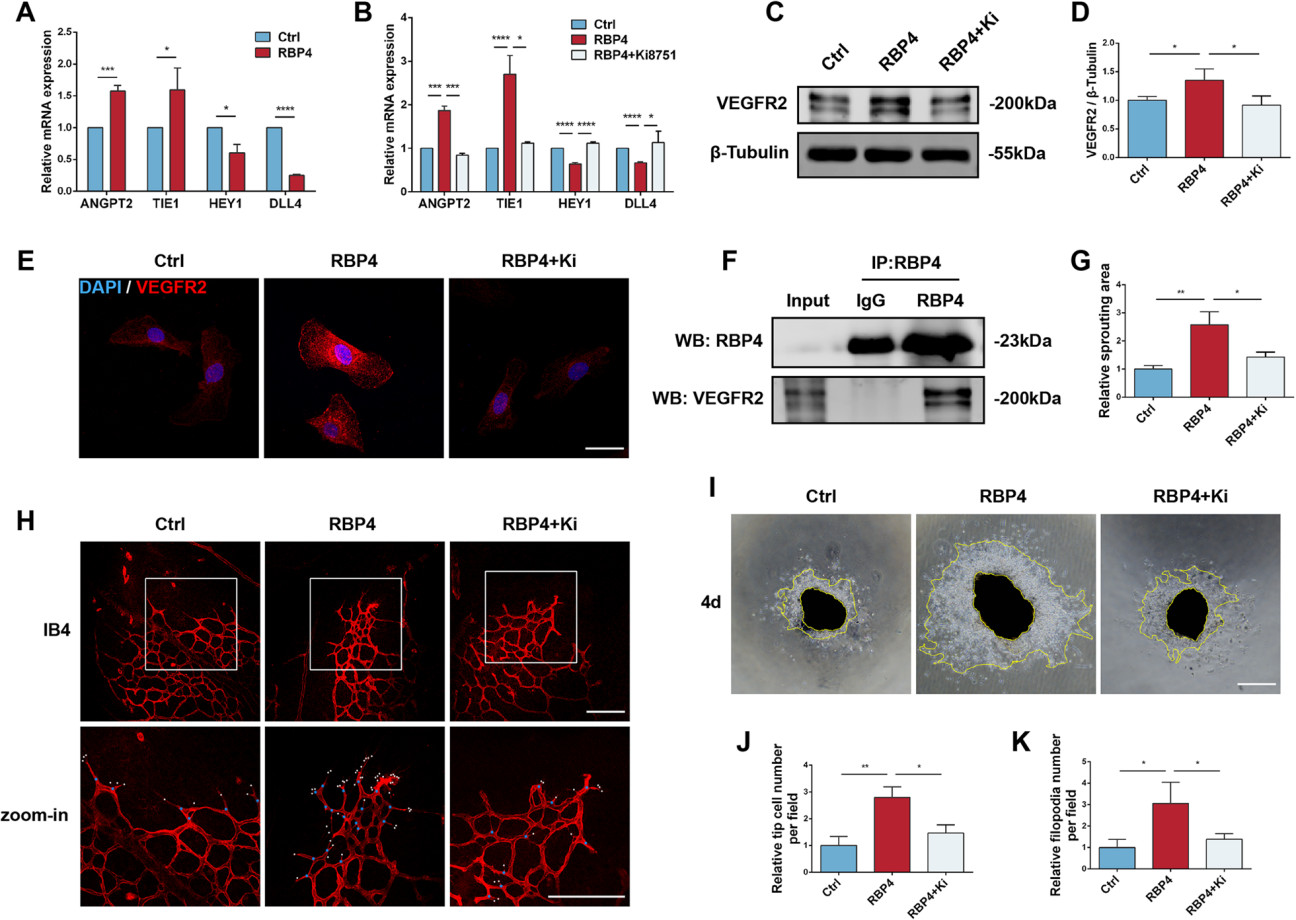
Effect of RBP4 on sprouting in vivo and in vitro. HUVECs were treated with 10 ng/ml RBP4 for 48 h, with or without 1 μM Ki8751 pretreatment for 12 h. **A** Relative mRNA expression of ANGPT2, TIE1, HEY1 and DLL4 in RBP4-treated HUVECs (*n* = 3). **B** Relative mRNA expression of ANGPT2, TIE1, HEY1 and DLL4 in the control, RBP4 and RBP4 + Ki8751 groups (*n* = 3). **C**, **D** Protein levels of VEGFR2 in the three groups (*n* = 3). **E** Immunofluorescence staining of DAPI (blue) and VEGFR2 (red) in HUVECs. Scale bar: 50 μm. **F** Co-IP examination of VEGFR2-RBP4 interaction. **H**, **J**, **K** Retinal IB4 staining of P14 in OIR and relative number of tip cells as well as filopodia. Scale bar: 200 μm (*n* = 3). **G**, **I** Choroidal sprouting analysis in three groups (*n* = 3). Scale bar: 500 μm. *P < 0.05, **P < 0.01, ***P < 0.001, ****P < 0.0001

Angiogenesis is mainly driven by endothelial tip cells, and to investigate whether RBP4 regulates vascular endothelial sprouting, we examined the expression of tip cell-related genes. In HUVECs, treatment with recombinant RBP4 protein (10 ng/mL for 48 h, #HEK293, MCE) resulted in upregulated expression of tip cell-enriched genes (ANGPT2 and TIE1) and downregulation of stalk cell-enriched genes (HEY1 and DLL4, Fig. 8A). VEGFR2, a tip cell-specific marker [20, 41], was increased in HUVECs after RBP4 intervention and was enriched in the cell membrane and cytoplasm (Fig. 8C–E). Administration of the VEGFR2 inhibitor Ki8751 (1 μM for 12 h, #HY-12038, MCE) reduced VEGFR2 expression and attenuated RBP4 promotion of ANGPT2 and TIE1 as well as inhibition of HEY1 and DLL4 (Fig. 8B). To further confirm the interaction of VEGFR2 with exogenous RBP4, we performed Co-IP assays. Using an RBP4 antibody, HUVECs showed strong binding to exogenous RBP4 protein, which indicated that the RBP4–VEGFR2 interaction may play a crucial role in tip cell specialization (Fig. 8F).

In the RNV model, the results demonstrated that administration of RBP4 induced an increased number of vascular tip cells and filopodia, while decreased retinal vascular sprouting after VEGFR2 inhibition was detected compared with retinal samples in P14 OIR (Fig. 8H, J, K). In advance, the isolated choroidal explants revealed that RBP4 increased the choroidal sprouting area on day 4 in comparison to untreated explants, whereas the sprouting area was reduced after Ki8751 intervention (Fig. 8G, I). These findings demonstrate that RBP4 modulates the tip phenotype of ECs through VEGFR2, thereby promoting neovascularization.

## Discussion

The effects of Mφs on tip cell specification-related neovascularization remain unclear. The current study demonstrated that succinate could modify the biofunctions of Mφs, thus promoting tip cell formation and angiogenesis. Succinate is an intermediate product of the adenosine TCA cycle and plays a crucial role in mitochondrial adenosine triphosphate (ATP) production. Abnormalities in mitochondrial metabolism may lead to the accumulation of succinate in the cytoplasm and/or the extracellular space. Previous research has shown that succinate is detected in body fluid samples and acts as a metabolic signal for local stress and immune risk by activating HIF-1α-dependent genes [42]. In the intraocular environment, Matsumoto et al. found a significant increase in succinate in the vitreous fluid of patients with PDR [43]. The metabolite changes in AH were investigated in our study. In addition to being actively secreted by the ciliary epithelium, AH is capable of being generated from the blood by diffusion and ultrafiltration. It circulates through the intraocular space and flows out of the eye into the venous blood by its continuous formation [44]. Thus, AH samples may serve as an important medium to reflect intraocular metabolism. Our results show that the TCA cycle is abnormally active in the AH of wAMD patients and that the decrease in pyruvate may be due to the enhanced TCA cycle depletion of downstream substrates. Changes in succinate in AH may be associated with altered oxygen demand and mitochondrial reserve capacity in the retina, ciliary body, and iris, thus succinate could be regarded as potential biomarkers in advanced studies.

Although alterations in succinate have been observed in several ocular diseases, the specific mechanism of succinate function in CNV and RNV has not been investigated. In our study, colocalization of both SUNCR1 and the M2 marker Arg1 in both CNV and OIR models demonstrated that succinate could regulate Mφ polarization. It has been reported that cells sense extracellular succinate through SUNCR1 and that the succinate–SUCNR1 signaling axis participates in a complex manner in immune responses. Succinate accumulates in immune cells, thereby stabilizing HIF-1α or sending inflammatory signals through SUCNR1 [45]. Exogenous succinate could trigger a local proinflammatory phenotype in myeloid cell-specific SUCNR1-deficient mice and disrupt glucose metabolism in normal diet-fed mice [46]. In addition, succinate secreted by cancer cells was able to convert Mφs into M2-polarized tumor-associated Mφs [33].

In this study, we demonstrated that succinate supplementation resulted in an increase in the proportion of Mφs with M2 polarization, accompanied by greater proliferation, migration, and tube-forming capacity of vascular endothelial cells, as well as more severe neonatal abnormal blood vessels in CNV and OIR models. The M1 and M2 types are two activation states of Mφs. Due to the varying environment, Mφs maintain a dynamic balance between these two polarizations [47]. Mφs have been widely discussed in CNV and RNV because of their critical regulatory role in inflammation and neovascularization [7, 48]. Paradoxically, however, studies exist in which Mφs both promote and inhibit the development of neovascularization, suggesting that the polarized phenotype of Mφs may be more relevant than the absolute quantity of infiltrates [3, 49]. Zandi et al. demonstrated that local injections of M2-type Mφs could exacerbate the severity of CNV, while M1-type Mφs showed the opposite effect [50]. In our model of CNV, both types of Mφs are detected, and the increase in M2 polarization is not necessarily accompanied by a decrease in M1-type Mφs, which may remain unchanged or increase slightly. Therefore, we speculated that in the complex intraocular environment, Mφ phenotypes are altered during disease development, and the increased M2/M1 ratio may tend to induce the formation of abnormal neovascularization.

In addition, both bone marrow-derived macrophages (BMs) and peritoneal macrophages (PMs) are widely used in studies related to the immune environment in ocular disease research [51, 52]. It stands to reason that BMs are more compatible with physiologic processes. While in comparison with PMs, which were cultured in vitro for only 1 day, BMs stimulated with M-CSF of for 7 days exhibited higher expression of TGF-β and IL-10, implying that BMs are more likely to have an M2 phenotype, as well as more variable in terms of maturation and phenotypic stability than PMs [53]. Furthermore, due to our large demand for PMs and the requirement for comparatively long interventions, we chose to culture PMs, which were easier to obtain and intervene. Although it has been suggested that different types of thioglycollate may extract PMs with variable conversion of Mφ states [54], referring to related studies [33], we selected the more commonly used thioglycollate to ensure the referability of the cells. Unfortunately, whether there is a difference in the effect of succinate on PMs and BMs was not investigated in this article and deserves further exploration in subsequent studies.

Given the proximity of Mφs to vascular tissues, we explored whether Mφs could directly and locally deliver information in a paracrine manner. In combination with RNA-Seq and in vitro validation, RBP4, which belongs to the lipid transport protein family and is a principal transporter protein of the circulating hydrophobic molecule retinol [55], acted as a novel secretory factor related to neovascularization after succinate intervention in Mφs. Although many of the functions of RBP4 depend on its role in retinol homeostasis, studies have described its function independently of retinol transport. RBP4 in adipose tissue induces an inflammatory response by the immune system, particularly by antigen-presenting cells such as dendritic cells, Mφs and CD4 T cells, which activate TLR2/4 [56, 57]. This inflammatory response is correlated with the c-Jun N-terminal kinase of RBP4 and is independent of the correlation with retinol [58]. RBP4 levels have been strongly associated with cardiovascular disease. Recent studies have reported that circulating RBP4 levels were increased in individuals with established carotid atherosclerosis and were related to the severity of stenosis [59]. RBP4 enhances the metastatic potential of breast cancer tumors through direct action on cancer cells and by increasing endothelial dysfunction and vascular damage within the tumor [60]. In summary, RBP4 was identified as a novel pro-angiogenetic factor as well as a linker between Mφ pathology and neovascularization.

In this study, we linked RBP4 and tip cell formation, the vanguard of angiogenesis, to investigate the specific mechanism of RBP4 in neovascularization. Interestingly, it has been reported that RBP4 levels were elevated in the vitreous of patients with PDR disease and decreased after administration of anti-VEGF therapy, further demonstrating the potential value of RBP4 in RNV [61]. However, more complex mechanisms involving metabolism, immunity, neovascularization and fibrosis may remain to be elucidated given the unique pathologic state of diabetes. Vascular sprouting depends on the recruitment of ECs into subtypes with specialized functions. In vivo, migratory tip cells extend long filopodia and act as navigators, but mitosis is quiescent [62]. Stem cells then proliferate to extend the branches. Once the vessels are perfused, the ECs become mature phalanx cells [63]. However, even though tip cell specification is a genetically conserved process in angiogenesis, detailed biomarkers of tip cells remain limited, thus prohibiting the understanding of RBP4 in tip cell formation.

Tip cells differ from more proximal proliferating stem cells and phalanx cells in several ways and express different genes. Internalized VEGFR2 is essential for the development of angiogenesis in vivo. RIN2/Rab5C was found to stabilize VEGFR2 expression via the ERK and PI3-K pathways, mediating tip cell specialization during angiogenesis [41]. Our results show that RBP4 activates VEGFR2 expression while promoting the levels of ANGPT2 and TIE, two known tip cell-positive related genes. ANGPT2 belongs to the endothelial growth factor angiopoietin family and is significantly upregulated at sites of vascular remodeling [64]. Mechanistically, ANGPT2 can act on the cytoskeleton of ECs to induce migration [65]. Knockdown of ANGPT2 leads to a lack of tip cells in the anterior part of neointimal sprouts in the mouse retina [66]. The orphan receptor Tie1 expressed by angiogenic ECs participates in the formation of the tip cell phenotype by downregulating Tie2 [67]. Meanwhile, RBP4 intervention could also negatively regulate DLL4 and HEY1 via VEGFR2, which is consistent with the inhibitory effects of DLL4 and HEY1 on tip cells in a previous report [68, 69]. In conclusion, there exists a potential link between RBP4 and EC sprouting, while the specific mechanism of regulation of VEGFR2 mediated by RBP4 deserves further investigation.

## Conclusion

In summary, we provide significant evidence that succinate accumulation due to metabolic abnormalities is a key regulator of the Mφ phenotype and pathological neovascularization. Succinate mediates the M2 polarization of Mφs via SUCNR1 and enhances the proliferation, migration, and tube-forming capacity of vascular ECs. In addition, the secretion of RBP4 stimulated by succinate-induced Mφs activated VEGFR2 and targeted the specialization of tip cells to facilitate angiogenesis. These novel findings identify succinate as a key proangiogenic factor that may provide a valuable diagnostic or therapeutic target for ocular vascular disease.

### Supplementary Information

**Additional file 1: Table S1.** List of primer information. **Figure S1. **Basic information for patients with CAT and wAMD. (A) Age, gender and IOP information in two groups (*n*=5). (B) Typical fundus photographs and OCT of patients. **Figure S2. **FA images at day 7, 14 and 21 after Matrigel intervention and control groups in CNV.** Figure S3. **Protein levels in Mφs after induction with different concentrations of succinate (0, 0.5 mM, 1 mM, and 2 mM for 48 h) by ELISA analysis. (A) Protein levels of TNFα (n=3). (B) Protein levels of iNOS (n=3). (C) Protein levels of IL6 (n=3). *P<0.05, **P<0.01, ***P<0.001. **Figure S4. **Results of wound healing and Transwell assays of HUVECs. HUVECs were intervented with hydroxyurea (200 mM, 24h) and co-cultured with Mφs (pretreated with siNC and 1mM succinate), being divided into three groups (siNC, siNC+ hydroxyurea, siNC+hydroxyurea+succinate). (A, B) Images were measured at 0 h, 6 h, 12 h and 24 h in the wound healing test. The cell migration rate was used to indicate migratory ability as described in the article. Scale bar: 200 μm (*n*=3). (C, D) In the Transwell assay, ImageJ software was used to calculate the stained cells in three groups. Scale bar: 200 μm (*n*=3). *P<0.05, **P<0.01.** Figure S5. **Protein levels of VEGFR2 and MMP2 in HUVECs co-cultured with four groups of Mφs (siNC, siNC+succinate, siSUCNR1 and siSUCNR1+succinate).(A) WB bands of VEGFR2, MMP2 and β-Tubulin in four groups. (B) Relative protein levels of VEGFR2 in HUVECs (*n*=3). (C) Relative protein levels of MMP2 in four groups (*n*=3). *P<0.05, ***P<0.001. **Figure S6. **Waveform and amplitude of ERGs in four groups (control, OIR+shNC, OIR+shNC+succinate and OIR+shSUCNR1+succinate) (*n*=3). *P<0.05, **P<0.01, ***P<0.001.

## Data Availability

Data supporting the findings of this study are available upon reasonable request to the corresponding author.
