# Supplementary material for: Succinate-induced macrophage polarization and RBP4 secretion promote vascular sprouting in ocular neovascularization
Source: J Neuroinflammation. 2023 Dec 21;20:308. doi: 10.1186/s12974-023-02998-1 (PMC10734053; doi:10.1186/s12974-023-02998-1)
Supplement: Supplementary file 1 — Additional file 1: Table S1. List of primer information. Figure S1. Basic information for patients with CAT and wAMD. (A) Age, gender and IOP information in two groups (n=5). (B) Typical fundus photographs and OCT of patients. Figure S2. FA images at day 7, 14 and 21 after Matrigel intervention and control groups in CNV. Figure S3. Protein levels in Mφs after induction with different concentrations of succinate (0, 0.5 mM, 1 mM, and 2 mM for 48 h) by ELISA analysis. (A) Protein levels of TNFα (n=3). (B) Protein levels of iNOS (n=3). (C) Protein levels of IL6 (n=3). *P<0.05, **P<0.01, ***P<0.001. Figure S4. Results of wound healing and Transwell assays of HUVECs. HUVECs were intervented with hydroxyurea (200 mM, 24h) and co-cultured with Mφs (pretreated with siNC and 1mM succinate), being divided into three groups (siNC, siNC+ hydroxyurea, siNC+hydroxyurea+succinate). (A, B) Images were measured at 0 h, 6 h, 12 h and 24 h in the wound healing test. The cell migration rate was used to indicate migratory ability as described in the article. Scale bar: 200 μm (n=3). (C, D) In the Transwell assay, ImageJ software was used to calculate the stained cells in three groups. Scale bar: 200 μm (n=3). *P<0.05, **P<0.01. Figure S5. Protein levels of VEGFR2 and MMP2 in HUVECs co-cultured with four groups of Mφs (siNC, siNC+succinate, siSUCNR1 and siSUCNR1+succinate).(A) WB bands of VEGFR2, MMP2 and β-Tubulin in four groups. (B) Relative protein levels of VEGFR2 in HUVECs (n=3). (C) Relative protein levels of MMP2 in four groups (n=3). *P<0.05, ***P<0.001. Figure S6. Waveform and amplitude of ERGs in four groups (control, OIR+shNC, OIR+shNC+succinate and OIR+shSUCNR1+succinate) (n=3). *P<0.05, **P<0.01, ***P<0.001. [file 12974_2023_2998_MOESM1_ESM.pdf]

Additional file 1

Table S1. List of primer information.

Gene name	Forward (5'→ 3')	Reverse (5'→ 3')
mouse-SUCNR1	GGCAGAGTTTTCTGTCGAGAC	ACATTCCCAAGCAGTCCAA
mouse-TNFα	CAGGCGGTGCCTATGTCTC	CGATCACCCCGAAGTTCAGTAG
mouse-iNOS	ACATCGACCCGTCCACAGTAT	CAGAGGGGTAGGCTTGTCTC
mouse-IL6	CTGCAAGAGACTTCCATCCAG	AGTGGTATAGACAGGTCTGTTGG
mouse-TGF-β	CCACCTGCAAGACCATCGAC	CTGGCGAGCCTTAGTTTGGAC
mouse-CD206	AAGGCGGTGACCTCACAAG	AAAGTCCAATTCCTCGATGGTG
mouse-Arg1	CCACAGTCTGGCAGTTGGAAG	GGTTGTCAGGGGAGTGTTGATG
mouse-RBP4	ACGAGTCCGTCTTCTGAGCA	TGGTCATCGTTTCCTCGCTG
human-ANGPT2	AACTTTCGGAAGAGCATGGAC	CGAGTCATCGTATTCGAGCGG
human-TIE1	ACGACCATGACGGCGAATG	CGGCAGCCTGATATGCCTG
human-HEY1	GTTCGGCTCTAGGTTCCATGT	CGTCGGCGCTTCTCAATTATTC
human-DLL4	GTCTCCACGCCGGTATTGG	CAGGTGAAATTGAAGGGCAGT
mouse-GAPDH	AGGTCGGTGTGAACGGATTTG	GGGGTCGTTGATGGCAACA
human-GAPDH	GGAGCGAGATCCCTCCAAAAT	GGCTGTTGTCATACTTCTCATGG

A

	CAT	wAMD	*P* value
Age	68.60 ± 7.06	70.40 ± 5.54	0.699
Male/Female	1/4	2/3	0.500
IOP	16.36 ± 1.29	14.92 ± 1.76	0.224

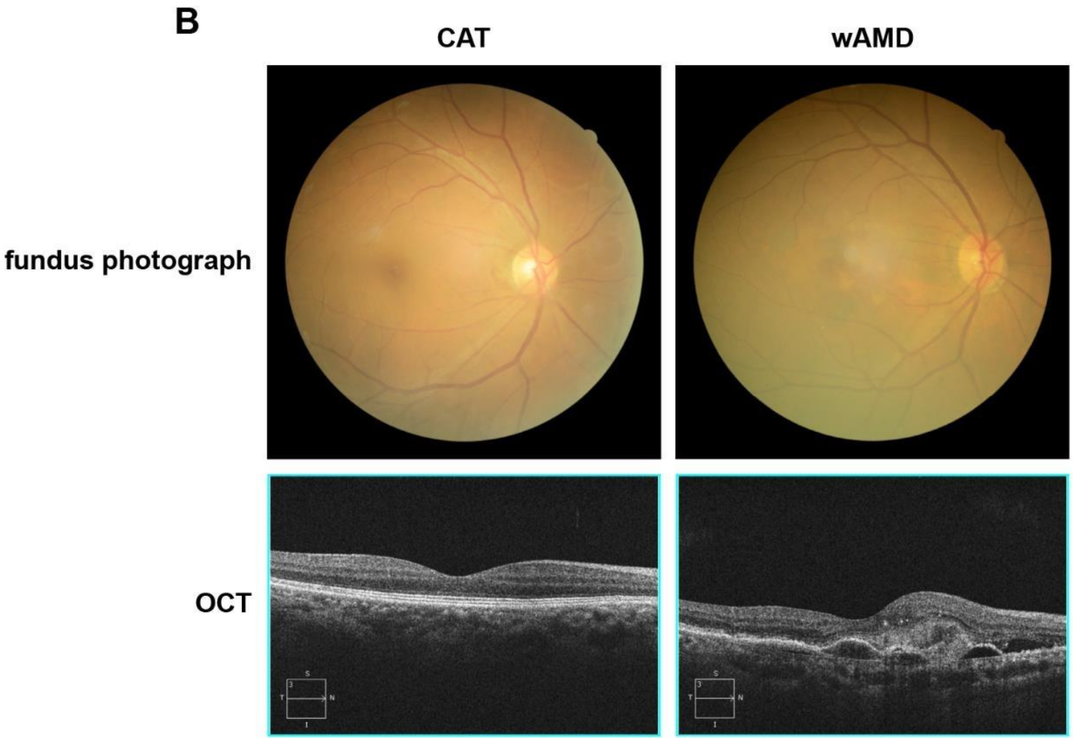

Figure S1. Basic information for patients with CAT and wAMD. (A) Age, gender and IOP information in two groups (*n*=5). (B) Typical fundus photographs and OCT of patients.

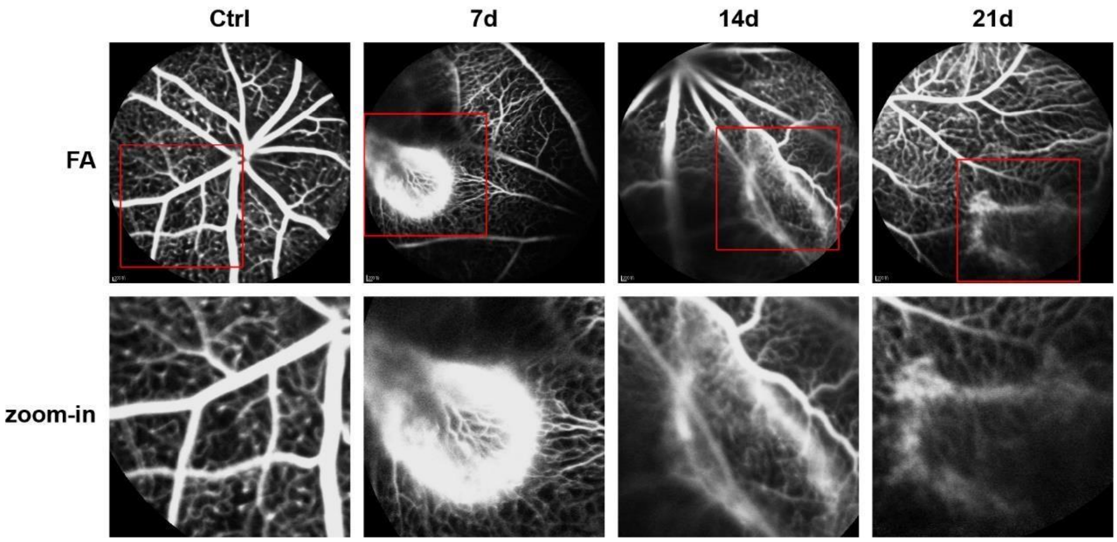

Figure S2. FA images at day 7, 14 and 21 after Matrigel intervention and control groups in CNV.

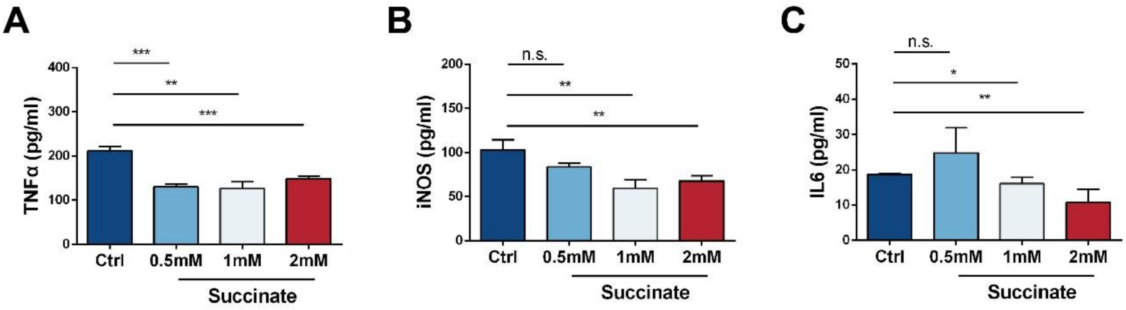

Figure S3. Protein levels in Mφs after induction with different concentrations of succinate (0, 0.5 mM, 1 mM, and 2 mM for 48 h) by ELISA analysis. (A) Protein levels of TNFα (n=3). (B) Protein levels of iNOS (n=3). (C) Protein levels of IL6 (n=3).

**P*<0.05, ***P*<0.01, ****P*<0.001.

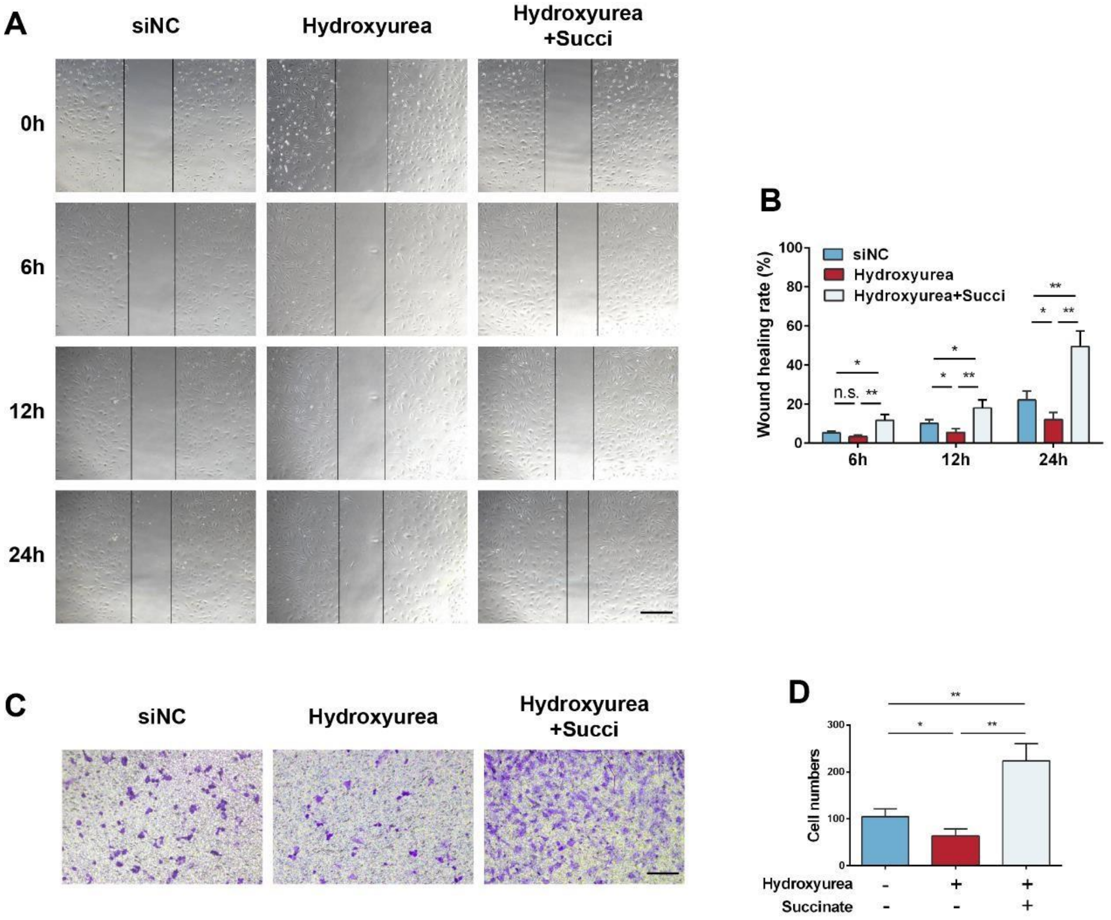

Figure S4. Results of wound healing and Transwell assays of HUVECs. HUVECs were intervented with hydroxyurea (200 mM, 24h) and co-cultured with Mφs (pretreated with siNC and 1mM succinate), being devided into three groups (siNC, siNC+ hydroxyurea, siNC+hydroxyurea+succinate). (A)(B) Images were measured at 0 h, 6 h, 12 h and 24 h in the wound healing test. The cell migration rate was used to indicate migratory ability as described in the article. Scale bar: 200 μm (*n*=3). (C)(D) In the Transwell assay, ImageJ software was used to calculate the stained cells in three groups. Scale bar: 200 μm (*n*=3). **P*<0.05, ***P*<0.01.

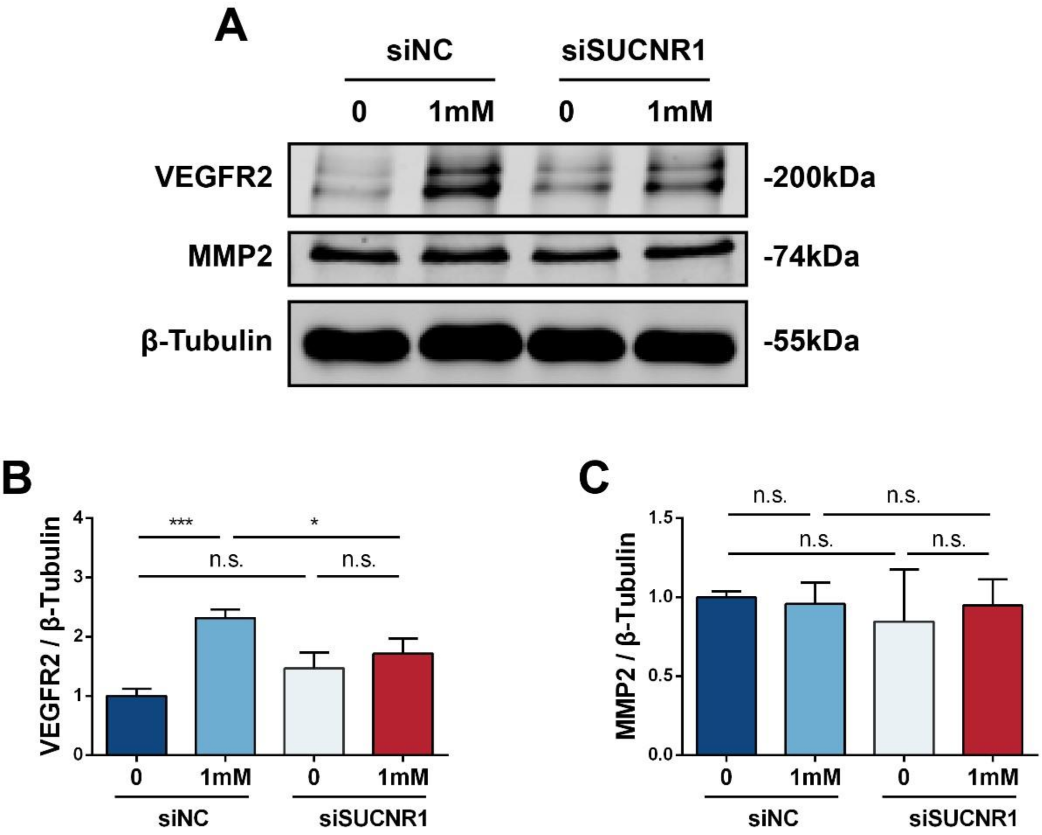

Figure S5. Protein levels of VEGFR2 and MMP2 in HUVECs co-cultured with four groups of Mφs (siNC, siNC+succinate, siSUCNR1 and siSUCNR1+succinate).(A) WB bands of VEGFR2, MMP2 and β-Tubulin in four groups. (B) Relative protein levels of VEGFR2 in HUVECs (*n*=3). (C) Relative protein levels of MMP2 in four groups (*n*=3). *P<0.05, ***P<0.001

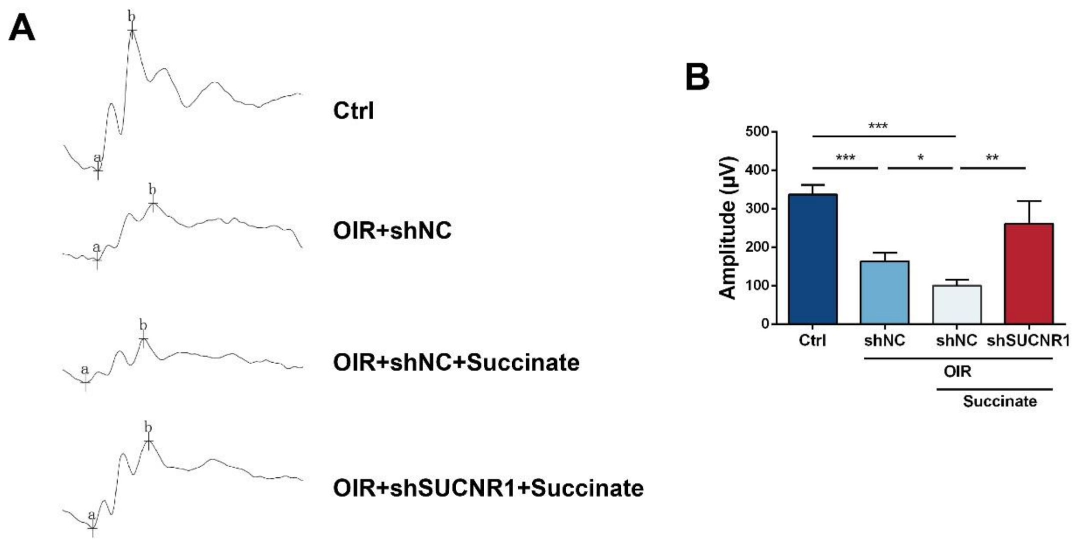

Figure S6. Waveform and amplitude of ERGs in four groups (control, OIR+shNC, OIR+shNC+succinate and OIR+shSUCNR1+succinate) (n=3). **P*<0.05, ***P*<0.01, ****P*<0.001.
